# Plant community composition and species richness in the High Arctic tundra: From the present to the future

**DOI:** 10.1002/ece3.3496

**Published:** 2017-10-25

**Authors:** Jacob Nabe‐Nielsen, Signe Normand, Francis K. C. Hui, Lærke Stewart, Christian Bay, Louise I. Nabe‐Nielsen, Niels Martin Schmidt

**Affiliations:** ^1^ Department of Bioscience Aarhus University Roskilde Denmark; ^2^ Arctic Research Centre (ARC) Aarhus University Aarhus C Denmark; ^3^ Department of Bioscience Aarhus University Aarhus C Denmark; ^4^ Mathematical Sciences Institute The Australian National University Acton ACT Australia

**Keywords:** Arctic tundra vegetation, climate change, latent variable models, Northeast Greenland, plant community composition, vascular plant species richness

## Abstract

Arctic plant communities are altered by climate changes. The magnitude of these alterations depends on whether species distributions are determined by macroclimatic conditions, by factors related to local topography, or by biotic interactions. Our current understanding of the relative importance of these conditions is limited due to the scarcity of studies, especially in the High Arctic. We investigated variations in vascular plant community composition and species richness based on 288 plots distributed on three sites along a coast‐inland gradient in Northeast Greenland using a stratified random design. We used an information theoretic approach to determine whether variations in species richness were best explained by macroclimate, by factors related to local topography (including soil water) or by plant‐plant interactions. Latent variable models were used to explain patterns in plant community composition. Species richness was mainly determined by variations in soil water content, which explained 35% of the variation, and to a minor degree by other variables related to topography. Species richness was not directly related to macroclimate. Latent variable models showed that 23.0% of the variation in community composition was explained by variables related to topography, while distance to the inland ice explained an additional 6.4 %. This indicates that some species are associated with environmental conditions found in only some parts of the coast–inland gradient. Inclusion of macroclimatic variation increased the model's explanatory power by 4.2%. Our results suggest that the main impact of climate changes in the High Arctic will be mediated by their influence on local soil water conditions. Increasing temperatures are likely to cause higher evaporation rates and alter the distribution of late‐melting snow patches. This will have little impact on landscape‐scale diversity if plants are able to redistribute locally to remain in areas with sufficient soil water.

## 1 INTRODUCTION

The response of Arctic plant communities to future climate changes depends on the degree to which their composition is determined by large‐scale climatic variations or by local environmental conditions. Increasing temperatures have been documented to influence community composition in some regions (Chapin, Shaver, Giblin, Nadelhoffer, & Laundre, 1995; Elmendorf et al., 2012b; Walker et al., 2006), frequently by enabling shrubs to become more dominant (Elmendorf et al., 2012b; Myers‐Smith et al., 2015). Such direct effects of climatic variations are less important when community composition is predominantly determined by local factors, such as topography or soil conditions, as habitats defined by fine‐scale variations may constitute refugia for some species and thereby buffer against effects of climate changes (Ackerly et al., 2010; Austin & Van Niel, 2011; Scherrer & Körner, 2011). A better understanding of the extent to which Arctic plants are influenced by environmental conditions that operate at different spatial and temporal scales is, therefore, critical in improving our ability to predict the impacts of climate changes.

Several types of fine‐scale environmental conditions are known to influence Arctic plant communities (Elberling et al., 2008). The presence of late‐melting snow patches may be particularly important, as it influences soil moisture during the growing season, a potential key driver of plant community composition and diversity in low‐energy systems (le Roux, Aalto, & Luoto, 2013). On steep slopes and in wind‐exposed areas, the ground is covered by a thin snow layer during winter, allowing only species that can tolerate the abrasive winds to persist (Elberling et al., 2008; Körner, 2003). Local topography also influences the amount of solar radiation, which in turn affects the timing of snowmelt and the length of the growing season. This is known to have a large impact on the composition of alpine plant communities (Galen & Stanton, 1995; Scherrer & Körner 2011). Some types of fine‐scale environmental conditions, such as the distribution of snow patches, will be influenced by climate change, while others will remain relatively constant. Plant communities associated with different types of fine‐scale environmental conditions are, therefore, expected to differ in their response to climate change.

Biotic interactions are another factor that contributes to structuring Arctic plant communities (Dormann & Brooker, 2002; Mod, Le Roux, & Luoto, 2014). In harsh environments, facilitation promotes species coexistence and in benign environments competition often reduces diversity (Gouhier, Menge, & Hacker, 2011; Michalet et al., 2006). These processes result in a humped‐back relationship between species richness and productivity (Michalet et al., 2006). Furthermore, increasing summer temperatures increase productivity and plant cover in the Arctic (Bhatt et al., 2010), which may make competition increasingly important in Arctic plant communities.

Community responses to climate change can be predicted using space‐for‐time substitutions, where statistical associations between extant community compositions and abiotic variables are used to define the set of environmental conditions required by each community (Blois, Williams, Fitzpatrick, Jackson, & Ferrier, 2013; Peterson et al., 2011). This approach assumes that drivers of spatial gradients of species composition also drive temporal changes in diversity. The impact of climate changes predicted using space‐for‐time substitutions have been found to agree with those based on warming experiments and long‐term observations in the Arctic, except that they may overestimate the response to contemporary climate warming (Elmendorf et al., 2015). Coarse‐scale temperature and precipitation data from global climate models may often be inadequate for predicting impacts of climate change, particularly in rugged terrain where plants are associated with topographically defined microhabitats (Franklin et al., 2013). Here space‐for‐time substations are ideal as they can be used for detecting the relative importance of alternative fine‐scale drivers of plant community composition and species richness.

Although the response of Arctic plant communities to climate changes depends on whether plant distributions are determined by macroclimate or by fine‐scale environmental conditions and biotic interactions, our knowledge of the relative importance of the different types of environmental conditions is limited due to the scarcity of landscape‐scale studies based on randomly distributed plots, especially in the High Arctic. Here, we present the hitherto largest study of this kind from Greenland, based on data from three sites in High Arctic Northeast Greenland. We test (1) whether plant species richness is related to macroclimatic variation, as characterized by summer temperature and precipitation, by fine‐scale environmental factors related to topography or soil type, or by plant‐plant interactions. We also test (2) if plant cover is related to macroclimatic variation, as a positive relationship between cover and temperature could suggest that climate changes result in increased completion. Finally, we test (3) to what extent variations in plant community composition are related to macroclimate or to local environmental conditions. If plant community composition and diversity are influenced by macroclimatic variations, the communities will be directly affected by climate change. Otherwise, local environmental variation may buffer against impacts of climate change, depending on whether the local conditions are sensitive to climate changes.

## 2 MATERIALS AND METHODS

### 2.1 Study sites

The study was conducted at three sites along Young Sund in Northeast Greenland: Tyrolerfjord (74°27′N, 21°39′W), Zackenberg (74°28′N, 20°34′W), and Blæsedalen (74°15′N, 19°53′W). The sites were selected as far apart on a coast‐inland gradient as logistically possible, to test whether the drivers of community composition and species richness were the same in areas with different soil characteristics and at different distances from the inland ice. Young Sund is a High Arctic fjord that extends 80 km west from the Greenland Sea toward the inland ice. The growing season generally lasts from June to August, but varies considerably among years depending on the exact timing of snowmelt. In sheltered depressions where snow accumulates during winter, the last snow may not melt until late August. The mean temperature in July is 5.8°C and yearly precipitation is 261 mm (Hansen et al., 2008). Further details on the environmental conditions in the area can be found in Elberling et al. (2008).

**Figure 1:**
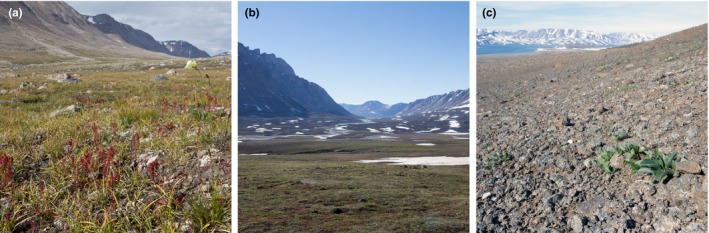
Typical vegetation in the three study sites in Young Sund, NE Greenland: (a) Tyrolerfjord, (b) Zackenberg, and (c) Blæsedalen

The vegetation in the study sites consists of a mosaic of vegetation types dominated by *Salix arctica*,* Dryas* sp. (cf. *D. integrifolia x octopetala*), and with patches of *Cassiope tetragona*. At the Tyrolerfjord and Zackenberg sites, poorly drained lowland areas are covered by dense fens dominated by *Eriophorum scheuchzeri* and *Dupontia fisheri*. At Zackenberg, lowland areas with moist soils are characterized by extensive grassland areas dominated by *Arctagrostis latifolia* and *Eriophorum triste* (Bay, 1998). At the Tyrolerfjord and Zackenberg sites, steep well‐drained upland slopes are dominated by *S. arctica* and *Dryas* sp. These are interspersed with very dry abrasion plateaus, where the sparse vegetation is dominated by *Kobresia myosuroides* and *Carex nardina*. Such plateaus occur in wind‐exposed areas that are nearly snow‐free year round. Blæsedalen is characterized by relatively small plants and very sparse vegetation cover even at low altitudes (Figure 1).

### 2.2 Experimental design

**Figure 2:**
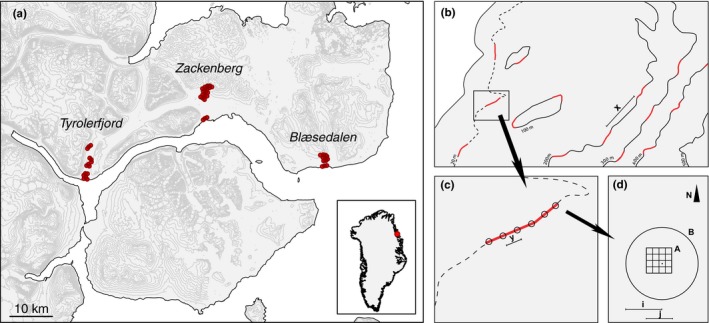
(a) Location of the 288 vegetation plots along Young Sund in Northeast Greenland. Contours show 100 m isoclines. (b) Distribution of plot groups (red lines) along isoclines within a study site. Three plot groups were selected for every 100 m increase in altitude, 500 m apart (=*x*). (c) Distribution of plots within a plot group (*y* = 10 m). (d) Placement of vegetation cover plots (A) and species inventory plots (B). Plots were permanently marked using metal poles (black dot). Contours in (a) are based on the GIMP DEM model (BPRC Glacier Dynamics Research Group, Ohio State University), and the coastline is based on data from the Danish Geodata Agency

In 2014–2015, 288 permanent plots were randomly selected across the three study sites in areas with gentle south, east, and west facing slopes. Three groups of six plots were selected for every 100 m increase in altitude (Figure 2). Plots were placed 10 m apart along the isoclines in areas with slope <45°. Neighboring plot groups were placed 500 m apart, except when the presence of steep slopes made it necessary to increase the distance slightly. On all sites, the lowest plots were placed 20 m above sea level (a.s.l.) to avoid direct salt exposure. The highest plots were placed 400 m a.s.l. in Tyrolerfjord (90 plots), 600 m a.s.l. in Zackenberg (126 plots), and 300 m a.s.l. in Blæsedalen (72 plots). Above these altitudes, the terrain was generally steep and barren.

The vegetation survey included a complete inventory of all species of vascular plants located <2 m from the plot centers. The vascular plant cover was assessed using the point‐intercept method (Walker, 1996) for the central part of the plots. We used a 0.7 m × 0.7 m point frame with 25 measurements spaced equidistantly within the frame. The fraction of the points that hit a vascular plant was used as a measure of plant cover for the plot. For pins that did not touch any plants, we recorded whether they hit a stone/rock (diameter >2 cm), water, or bare ground. Plants were identified following Böcher, Fredskild, Holmen, and Jakobsen (1978), but the nomenclature was subsequently updated based on the most relevant taxonomic databases (Table S1).

### 2.3 Environmental data

To assess the effects of fine‐scale environmental conditions on the plant communities, we recorded a number of environmental variables for each plot. Slope and aspect were recorded using a clinometer (Suunto, Finland) and a compass. Position and altitude were recorded using a GPS (GPSmap 62s; Garmin, USA), although the clinometer was used to ascertain that all plots in a plot group were located at the same altitude. Soil water content was recorded using a ML3 Theta Probe (Delta‐T Devices Ltd, UK) or a ProCheck (Decagon Devices, Inc.). We calculated the solar radiation index (Keating, Gogan, Vore, & Irby, 2007) based on the slope, aspect, and latitude of each plot.

To assess the influence of macroclimate on the plant communities, we calculated the monthly minimum, mean, and maximum temperatures, and monthly precipitation for each plot based on the WorldClim database version 1.4 (Hijmans, Cameron, Parra, Jones, & Jarvis, 2005; Fig. S1). The WorldClim climate variables were generated through interpolation of average monthly data from weather stations on a 30 arc‐second resolution (approx. 1 km^2^) for the period 1960–1990. They were modelled using thin‐plate smoothing with latitude, longitude, and elevation as independent variables (Hijmans et al., 2005). In the analyses below, we used average mean monthly temperature and precipitation for the months June–August to characterize climate in the plot groups.

To determine how differences in snow cover, number of hours with sunshine, soil depth etc. among the three study sites influenced community composition, we computed a continentality index. The index was calculated as the proportion of the distance moved between the outer coast and the inland glacier, which was characterized by a line running in the direction 22°, starting 7.5 km west of the westernmost plot. The easternmost plot defined the outer coast (Figure 2).

### 2.4 Statistical analyses

We used generalized linear mixed models to determine whether variations in plant species richness were best explained by temperature and precipitation (both representing macroclimate), altitude, slope, solar radiation, soil water (variables related to local topography and soil type), or by plant cover (representing plant‐plant interactions). Data were analzsed on the plot group level, as observations from individual plots were not considered independent. By aggregating observations from multiple plots, we reduced the influence of patchiness, which is notorious for High Arctic plants (Van der Wal & Stien, 2014). Therefore, the average number of species per plot in the plot groups was used as response variable. The average values of the macroclimatic and local environmental factors and plant cover in the plot groups were used as predictors. Study site was included as a random intercept in all models. To test if the number of species peaked for intermediate levels of plant cover, we included the “cover” variable as both a linear and a quadratic term. Because a preliminary inspection of the data suggested a non‐linear relationship between cover or number of species per plot and altitude, we included altitude as a categorical variable with seven levels (one for each isocline). All other predictor variables were continuous, and analyses of residuals from linear regressions suggested that both cover and number of species were linearly related to these variables, with no serious outliers. Furthermore, residuals were normally distributed and variances were homogeneous (Bartlett's test). The same method was used to determine which variables best explained variations in plant cover, but excluding plant cover as predictor.

To select the models that best explained variations in cover and number of species per plot, we calculated the corrected Akaike Information Criterion (AICc; Burnham & Anderson, 2002) for all possible models that included one or more predictor variables. We retained only models that had substantial empirical support (i.e. with ΔAIC_c_ < 2 relative to the model with lowest AIC_c_). Site was retained as random intercept in all models. We used Akaike weights (AW) to calculate the probability that each of the models was the best for the observed data (Johnson & Omland, 2004). The mixed models were fitted using the nlme package (Pinheiro, Bates, DebRoy, & Sarkar, 2016) in the statistical software R (R Development Core Team 2016).

To assess how plant community composition was influenced by environmental variation, we used latent variable models (LVMs; Hui, Taskinen, Pledger, Foster, & Warton, 2015; Warton et al., 2015). LVMs extend the generalized linear model framework to facilitate specification of joint statistical models for studying the abundance of many taxa. The presence/absence of all plant species in the plot groups was modelled simultaneously, assuming binomial responses with a probit link function. Only plants that were identified to species were included in the model (93% of all plants recorded), and only if they were among the 61 species that occurred in ≥5 plot groups (54% of the observed plant species). The covariates included the parameters identified to be important for species richness (i.e. average cover, slope, soil water, and solar radiation in the plot groups), as well as the continentality index variable. Importantly, the model also included two latent variables, which can be regarded as missing predictors that mediate residual correlations across taxa, that is variation which is not due to shared environmental response (Warton et al., 2015).

We quantified the extent to which the species covariation was explained by the five environmental covariates based on the change in marginal log‐likelihood relative to a model without these covariates (i.e. an unconstrained ordination with only the two latent variables included). The LVMs were fitted using the boral package (Hui, 2016) in R, which utilises Bayesian Markov chain Monte Carlo estimation performed via JAGS (Plummer, 2003). Models were fitted assuming weakly informative priors (Gelman, Jakulin, Pittau, & Su, 2008).

## 3 RESULTS

### 3.1 Environmental variability

Although temperature and precipitation varied among sites, the variation among plot groups within each site was generally larger (Fig. S1). The variability in temperatures within sites was mainly caused by a strong correlation between altitude and temperature (*r *= −0.82; Pearson correlation; Fig. S2). Temperature was strongly correlated with precipitation (*r *= −0.98). Maximum temperatures were >0°C in June–August, which is the main growing season on all sites.

The environmental parameters altitude, slope, solar radiation, and soil water varied considerably both among plot groups within each site and among sites (Fig. S2). Slopes were generally steeper on higher altitudes, and solar radiation was higher on steep slopes (*r *=* *0.66). Soil water was particularly strongly correlated with slope (*r *= −0.38), and most of the plot groups with high soil water content were found in the flat lowlands of Zackenberg and Tyrolerfjord. The average soil water level was considerably lower in Blæsedalen (17.9 ± 11.4%) than in Tyrolerfjord (31.0 ± 29.7%) or Zackenberg (31.4 ± 12.4%; mean ± 1*SD*), possibly because of a limited inflow of melt water from upland glaciers and late‐melting snow patches.

### 3.2 Variations in plant cover and species richness

**Figure 3:**
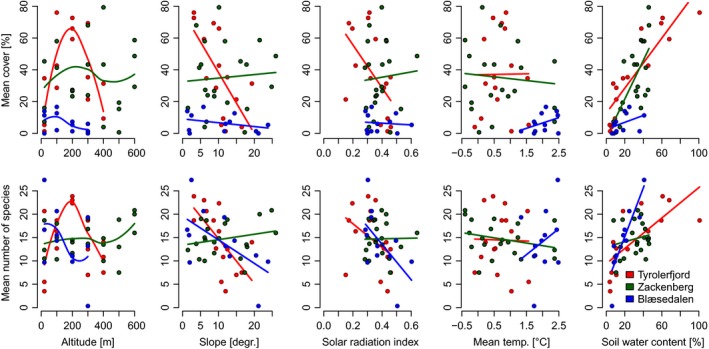
Relationships between mean plant cover and mean number of species per plot and the possible environmental drivers. Each line represents one of the three study sites. For altitude lowess fits were used, otherwise linear fits were used. Each point represents the average value for one plot group. Precipitation was highly correlated with summer temperature, and the corresponding figure is, therefore, omitted

Species richness was strongly related to soil water, which alone explained 35% of the variation in average number of species per plot (Table 1). It increased with increasing soil water in all sites (Figure 3). Three models that also included solar radiation, plant cover, or slope were also strongly supported by data. In the model that included plant cover, cover was included as both linear and quadratic terms, indicating a peak in species richness at intermediate cover (Fig. S3). The model that included only the linear term was, however, almost equally good (ΔAW < 0.01; *R* ^2^ **= 0.35). The climatic variables were not included in any of the four models that were supported by the Young Sund data. The best model that included at least one climatic variable had AIC_c_ **= 2.4 (AW* *= 0.04).

**Table 1:** Comparison of mixed models for explaining variations in mean cover and mean number of species per plot. Only the best models (ΔAIC_c_ < 2) are shown. AW are Akaike weights; *R* ^2^ is shown for fixed effects only as well as for the whole model, including study site

Response	Fixed variables	AIC_c_	ΔAIC_c_	AW	*R* ^2^ fixed	*R* ^2^ model
sp. count	Soil water	286.73	0.00	0.13	0.35	0.35
sp. count	Soil water + solar radiation	287.97	1.23	0.07	0.36	0.36
sp. count	Soil water + cover + cover sqr.	288.55	1.82	0.05	0.43	0.43
sp. count	Soil water + slope	288.63	1.90	0.05	0.35	0.35
Cover	Soil water + slope	−38.97	0.00	0.19	0.54	0.65
Cover	Soil water	−38.72	0.24	0.16	0.51	0.63
Cover	Soil water + summer precipitation	−38.11	0.87	0.12	0.60	0.63
Cover	Soil water + summer temperature	−38.07	0.91	0.12	0.61	0.63

Plant cover was also strongly related to variation in soil water, and this variable alone accounted for 51% of the variation in plant cover among the plot groups (Table 1). Jointly, soil water and site accounted for 63% of the variation in plant cover. Cover was highest on wet soils on all sites (Figure 3). Models that also included average slope, summer precipitation, or temperature were strongly supported by the data, but inclusion of these variables had only minor impact on the model's explanatory power. The relationship between cover and each of these variables differed among study sites. Among all possible models, the ones that did not include soil water were unlikely to explain variations in species richness (sum AW* *= 0.08).

### 3.3 Plant community responses to environmental variation

**Figure 4:**
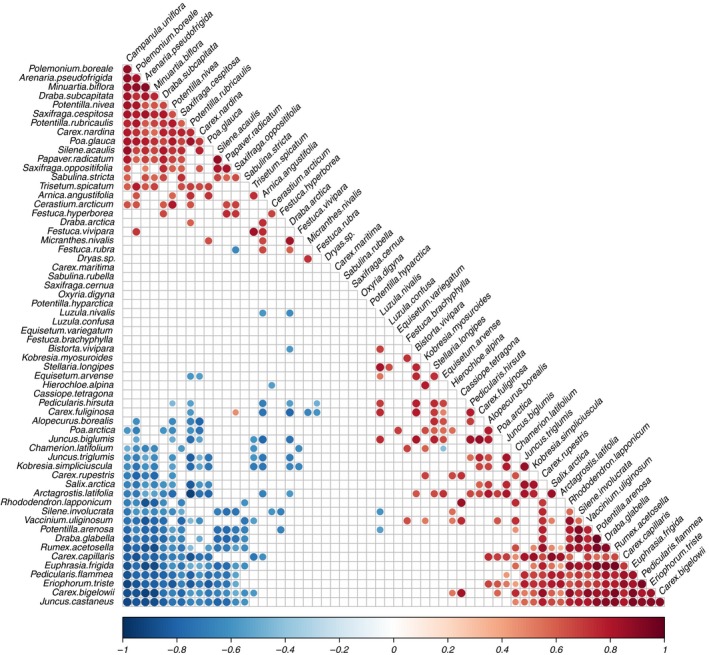
Species co‐occurrence patterns predicted using LVM model including the covariates cover, slope, soil water, solar radiation, continentality index, and two latent variables. Positive values (red) indicate that species are likely to occur in the same plot groups and negative values (blue) indicate that species are likely not to occur together. Only significant correlations are included in the plot, that is those whose 95% highest posterior interval does not contain zero

Approximately, half of the species in the analysis tended to co‐occur with one or more other species in plot groups with particular environmental conditions (Figure 4). Species at the top left of Figure 4 mostly occurred on dry soils in areas with low cover, far from the inland ice. Those to the right predominantly occurred on wet or moist soils at low altitudes at the most continental site. The species that co‐occurred with several other species were generally negatively associated with other groups of species. The species *Euphrasia frigida*,* Pedicularis flammea, Eriophorum triste*, and *Carex bigelowii,* for example, were likely to occur together in plot groups where, for example *Polemonium boreale* and *Arenaria pseudofrigida* were unlikely to occur. This grouping of species, as represented by significant positive and negative associations, resulted from their combined responses to cover, slope, soil water, solar radiation, and continentality.

Distributions of the individual species in the co‐occurring species groups were generally related to the same environmental covariates (Fig. S4). *Euphrasia frigida*,* Pedicularis flammea,* and *Eriophorum triste* were, for example, all positively related to soil water and continentality, whereas *Carex bigelowii* was only positively related to continentality (i.e. predominantly occurring inland). Several species, including the common species *Dryas* sp. and *Saxifraga cernua* (Table S2), did not appear to co‐occur with particular groups of species or to be associated with particular environmental covariates. Based on changes in log‐likelihood, the five environmental covariates jointly explained 29.4% of the species co‐occurrence patterns. In a similar analysis, where continentality was omitted as covariate, this value decreased to 23.0%. When continentality, temperature, precipitation, and the environmental covariates were all included in the model, it explained 33.7% of the species co‐occurrences.

## 4 DISCUSSION

### 4.1 Impacts of climate on species richness

Climate change is expected to influence plant diversity in the Arctic, but the nature of this effect is debated. Space‐for‐time substitutions predict that diversity will be strongly affected by climate changes if the most species rich communities are associated with particular macroclimatic conditions. This, however, did not appear to be the case in Young Sund. Here local species richness was unrelated to summer temperature and precipitation, variables that are generally considered important for arctic plants (Elmendorf et al., 2012a; Wahren, Walker, & Bret‐Harte, 2005). Instead, local species richness was mostly determined by variables related to topography, and soil water alone accounted for 35% of the variation in vascular plant species richness. Species richness was highest on moist soils at all study sites. These results suggest that the direct impact of climate change on local plant diversity will be negligible.

Although we found no evidence of a direct climate effect on plant species richness, increasing temperatures and changing precipitation patterns could have strong indirect impacts on the communities by influencing soil water. Soil water levels are strongly related to the distribution of late‐melting snow patches in the summer, as most precipitation in the study area falls as snow during the winter (Hansen et al., 2008). Increasing temperatures will result in earlier snow melt (Barnett, Adam, & Lettenmaier, 2005), causing the tundra to dry out earlier in summer. At the same time, increasing temperatures are expected to cause drying of soils in areas currently located close to late‐melting snow patches, which will ultimately result in decreased local diversity.

The absence of a direct effect of temperature on species richness contrasts with the findings in most experimental studies. A meta‐analysis from 11 different tundra localities showed increased cover of deciduous shrubs and graminoids as well as decreased species diversity in response to warming (Walker et al., 2006). Such decreasing species richness with increasing temperatures may be attributed to competitive exclusion of the less abundant species (Chapin et al., 1995; Wahren et al., 2005). Climate‐induced expansion of shrubs has been predicted to be most extensive in the “warm” parts of the Arctic tundra that are dominated by tall shrubs (Elmendorf et al., 2012a). The negligible effect of climate on plant diversity in Young Sund may, therefore, be attributable to the low cover of tall shrubs in the area.

Another possible reason for the small effects of climatic variations in Young Sund is the relatively small climatic gradient spanned in this study. In a study from Svalbard spanning a much larger climatic gradient, plant diversity was related to summer temperatures and precipitation (from WorldClim) as well as normalized deviation vegetation index (Nilsen, Arnesen, Joly, & Malnes, 2013). The temperatures vary between 1.2 and 2.8°C within each of the Young Sund study sites, and although this is a smaller gradient than the one in the Svalbard study, it is comparable to the temperature increase expected to take place in the region over the next four to six decades (Stendel, Christensen, & Petersen, 2008). Space‐for‐time substitutions, therefore, predict that the main effect of climate changes in this period will be mediated by their impacts on soil water contents and that the direct impacts of increasing temperatures will be negligible.

### 4.2 Relationship between plant cover and diversity

We found evidence that plant species richness was to some extent influenced by plant‐plant interactions, as one of the models that best explained variations in diversity indicated a peak in number of species for intermediate plant cover (Table 1; Fig. S3). The evidence was, however, weak, as a model that predicted a linear increase in species richness with cover was nearly as good. Although plant cover was likely related to temperature and precipitation, the effect differed among sites. Even if climate changes result in increased plant cover in some areas, our results do not suggest that this will cause marked increase in competition.

### 4.3 Influence of environmental variation on community composition

Plant community composition and species richness were to a large extent influenced by soil water, slope, plant cover, and solar radiation (Figure 4). In contrast to species richness, which was largely the same at the three study sites (Figure 3), the plant community composition was also influenced by distance to the inland ice. This was evidenced by the decrease in the explained part of the species co‐occurrence patterns from 29.4% to 23.0% when omitting the continentality index from the latent variable model. Inclusion of temperature and precipitation in the model caused it to explain 33.7% of the species co‐occurrences, suggesting that climate changes could have a direct, albeit minor, impact on plant community composition.

The most distinctive species groups identified by the model were associated with either wet soils (e.g. those including *Euphrasia frigida*,* Pedicularis flammea,* and *Eriophorum triste*) or inland sites (including e.g. *Arenaria pseudofrigida* and *Campanula uniflora*). Several of the species that were most likely to occur on wet soils were at the same time significantly associated with areas with relatively dense cover (e.g. *Arctagrostis latifolia, Equisetum arvense,* and *Eriophorum triste*; Fig. S4).

### 4.4 Effects on different spatial and temporal scales

At the spatial scale investigated in this study, soil moisture and other variables related to local topography had a strong impact on plant community composition and species richness. Community composition was also related to macro climatic variation within study sites and to differences among study sites, albeit only to a minor extent. This suggests that the distributions of some species are directly determined by large‐scale climatic variations and that climate changes could potentially enable new plant communities to establish in Young Sund. The direct effect of large‐scale climatic variability is, however, small, in comparison to the importance of fine‐scale environmental variability, which appears to largely buffer against any direct impacts of climate changes. Such buffering has previously been reported in non‐Arctic studies (Ackerly et al., 2010; Scherrer & Körner, 2011).

Our results strongly suggest that the main impact of climate changes on the High Arctic vegetation will be mediated by their influence on local soil water conditions. Increasing temperatures are likely to cause evaporation to increase and alter the distribution and overall cover of late‐melting snow patches. Although these negative impacts of increasing temperatures may to some extent be compensated for by increased summer precipitation (Collins et al. 2013; Stendel et al., 2008), precipitation alone is unlikely to ensure that the perpetually wet conditions that are currently found in the vicinity of late‐melting snow patches will be found in all parts of the landscape in the future. In some parts of the landscape, the plant communities associated with wet soils may, therefore, disappear, causing local diversity to decrease. As long as the soil moisture and topographic conditions that determine the composition of the present‐day plant communities still exist somewhere in the landscape, a decrease in the overall cover of late‐melting snow patches is, however, not likely to result in the disappearance of plant communities at the landscape scale.

The persistence of plant communities under climate change may be aided by microtopographic variability within the plot groups, and even within individual plots. This could enable plant communities to persist in a few microsites with favorable conditions in spite of generally deteriorating environmental conditions. Such microsite variability may be responsible for the slow change of plant communities observed on other Arctic sites during the past decades (Damgaard et al., 2016; Hudson & Henry, 2010; Jorgenson, Raynolds, Reynolds, & Benson, 2015). When climate changes occur at a pace that allows plant communities to redistribute along with the areas with favorable soil water conditions, or if microtopographic variability enables them to persist without redistributing, climate changes are, therefore, unlikely to result in the disappearance of plant communities or in decreased diversity at the landscape scale.

Whether our findings are representative for other parts of Greenland is an open question, as the local drivers of variations in plant species richness and community composition have not been studied on the landscape scale elsewhere. Studies that have found a direct effect of climate changes by means of controlled experiments were generally conducted on sites where the vegetation was denser and more dominated by shrubs than in Young Sund (Walker et al., 2006; Zamin, Bret‐Harte, & Grogan, 2014). In these studies, warming resulted in competitive exclusion of rare species by shrubs and in decreasing diversity. Our study indicates that High Arctic vegetation may respond differently to climate changes in being less prone to becoming dominated by shrubs, with plant communities that are more influenced by access to water and possibly local variability in other resources.

## CONFLICT OF INTEREST

None declared.

## AUTHOR'S CONTRIBUTIONS

JNN designed the study and led the writing of the manuscript. JNN, SN, LS, CB, and LNN conducted the fieldwork. JNN and FH analyzed the data. All authors contributed critically to the manuscript and gave final approval for publication.

## DATA ACCESSIBILITY

Data available from the Dryad Digital Repository: http://dx.doi.org/10.5061/dryad.8gr16 .
