# Supplementary material for: Plant community composition and species richness in the High Arctic tundra: From the present to the future
Source: Ecol Evol. 2017 Oct 25;7(23):10233–42. doi: 10.1002/ece3.3496 (PMC5723606; doi:10.1002/ece3.3496)
Supplement: Supplementary file 1 [file ECE3-7-10233-s001.pdf]

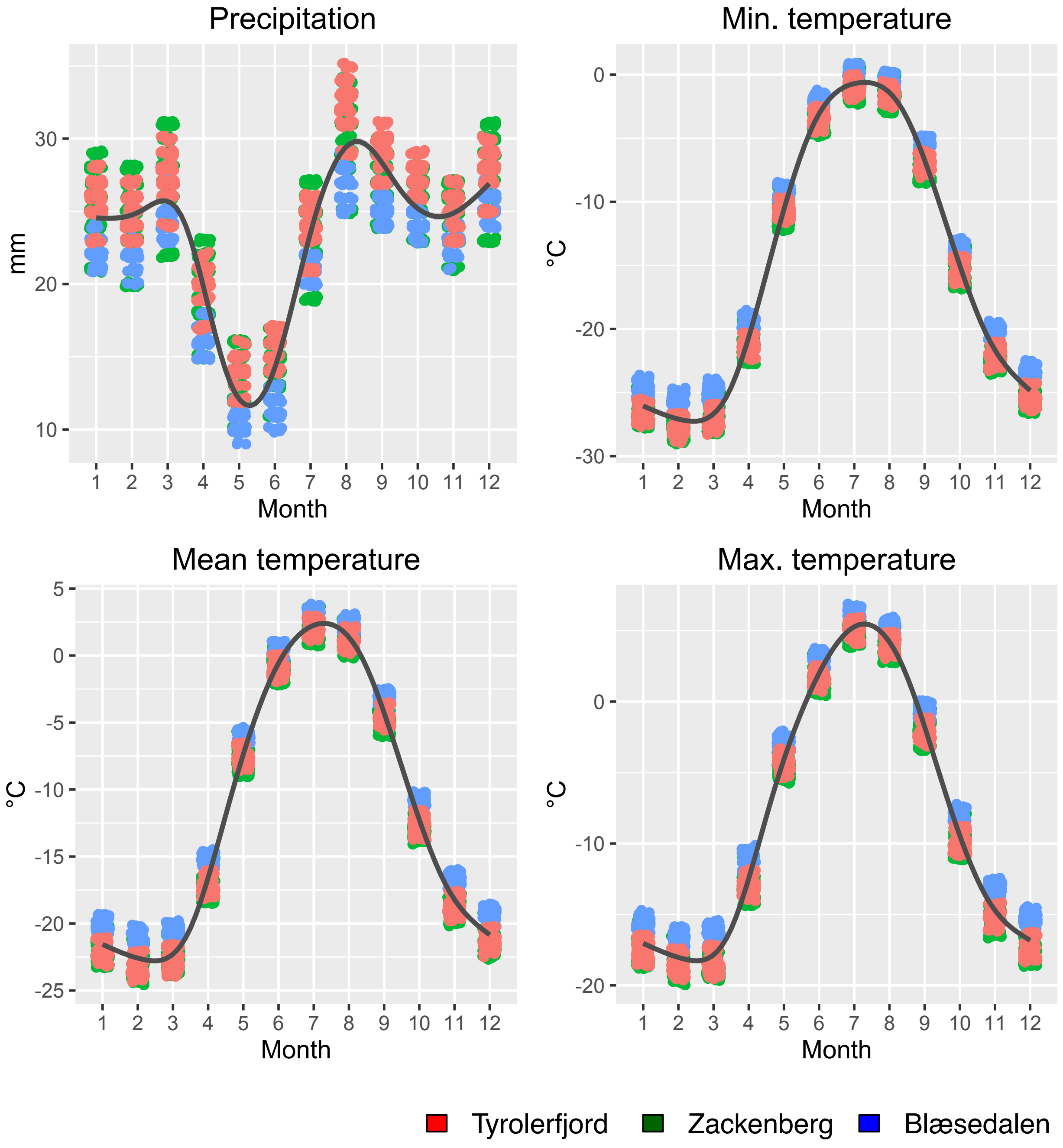
Precipitation
Min. temperature
Mean temperature
Max. temperature
mm
°C
Month
Tyrolerfjord
Zackenberg
Blæsedalen
