# Supplementary figures and images for: Plant community composition and species richness in the High Arctic tundra: From the present to the future
Source: Ecol Evol. 2017 Oct 25;7(23):10233–42. doi: 10.1002/ece3.3496 (PMC5723606; doi:10.1002/ece3.3496)

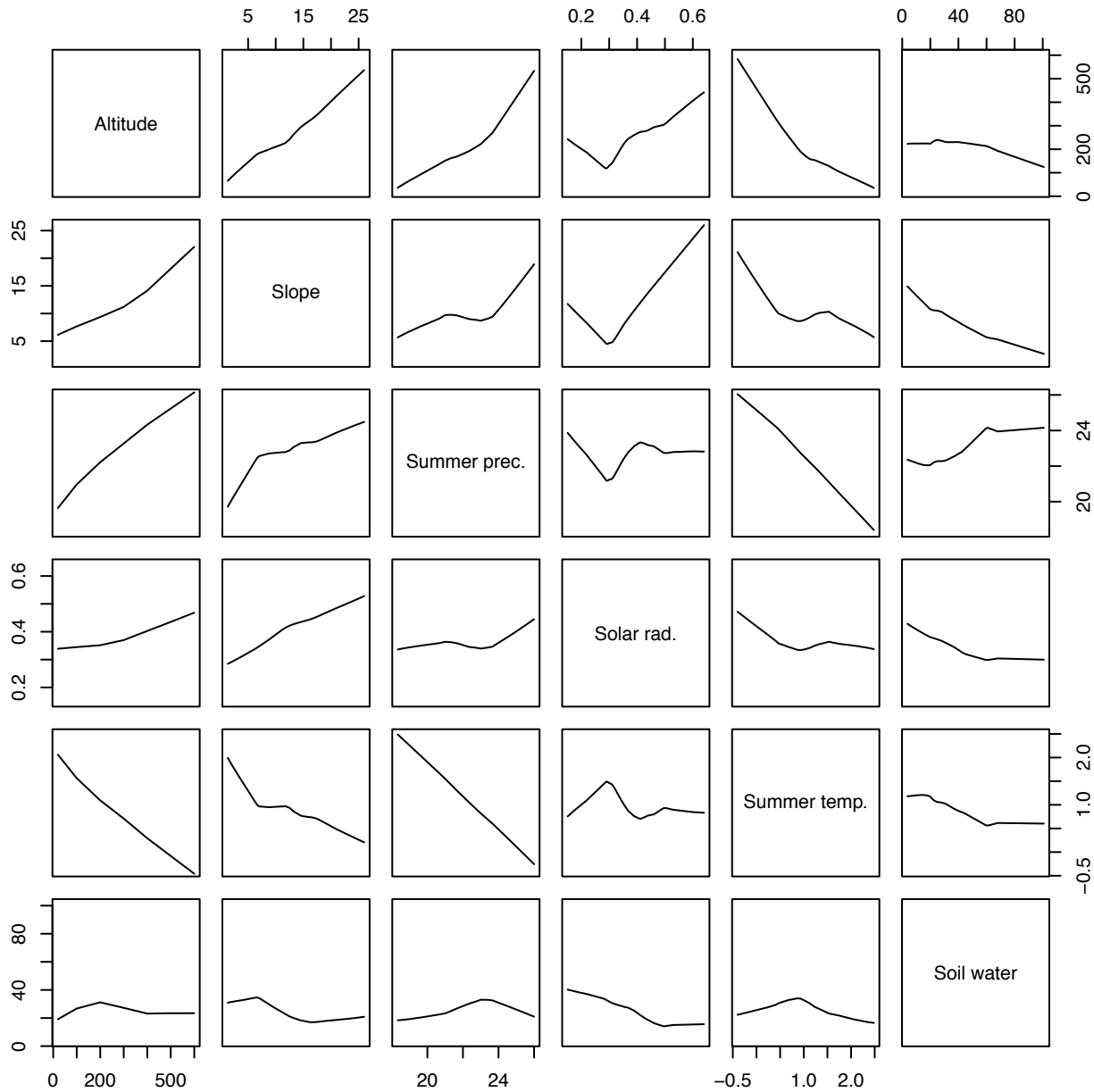

■ Tyrolerfjord
 ■ Zackenberg
 ■ Blåsedalen

Supplement: Supplementary file 2 [file ECE3-7-10233-s002.pdf]

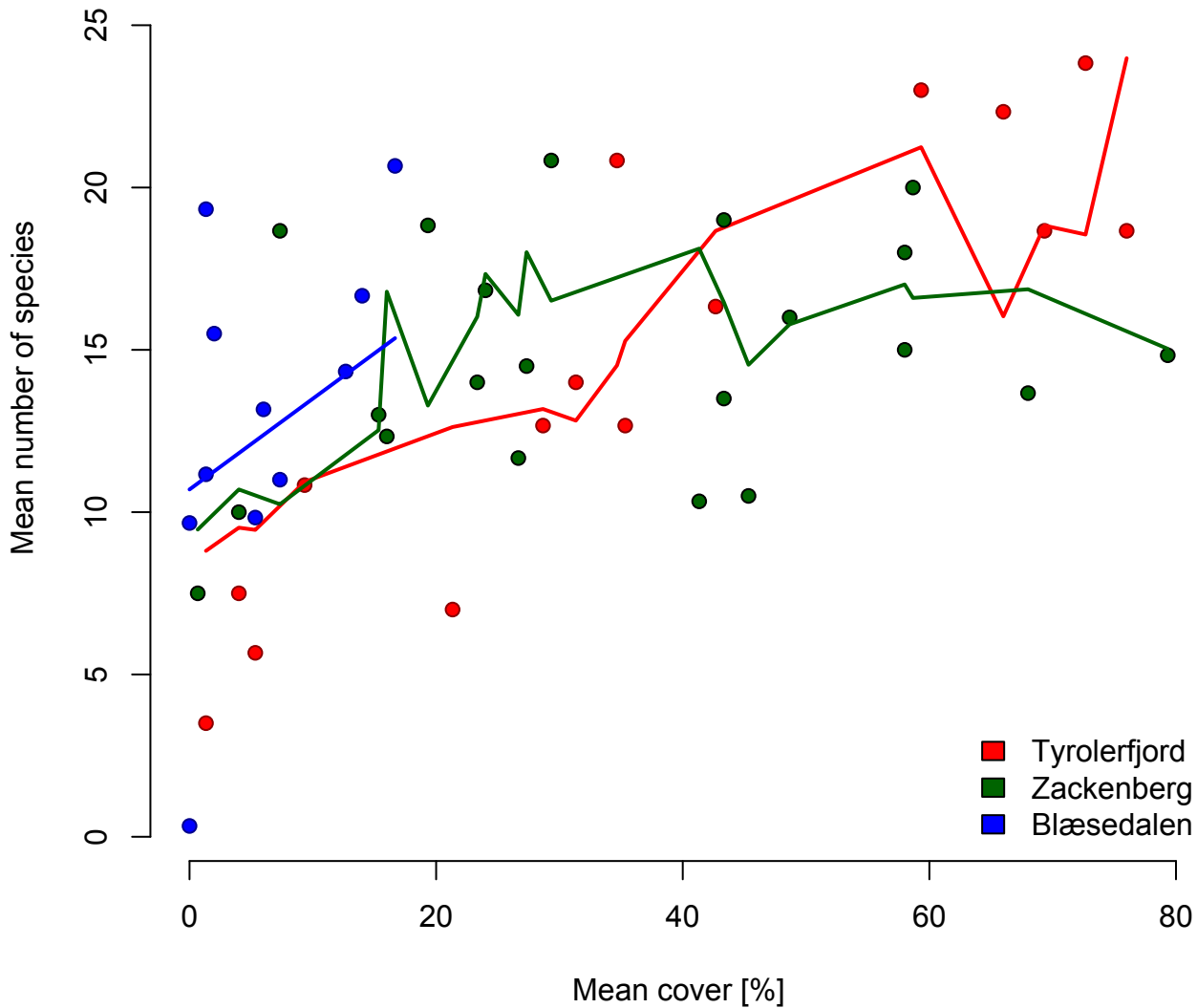

Supplement: Supplementary file 3 [file ECE3-7-10233-s003.pdf]

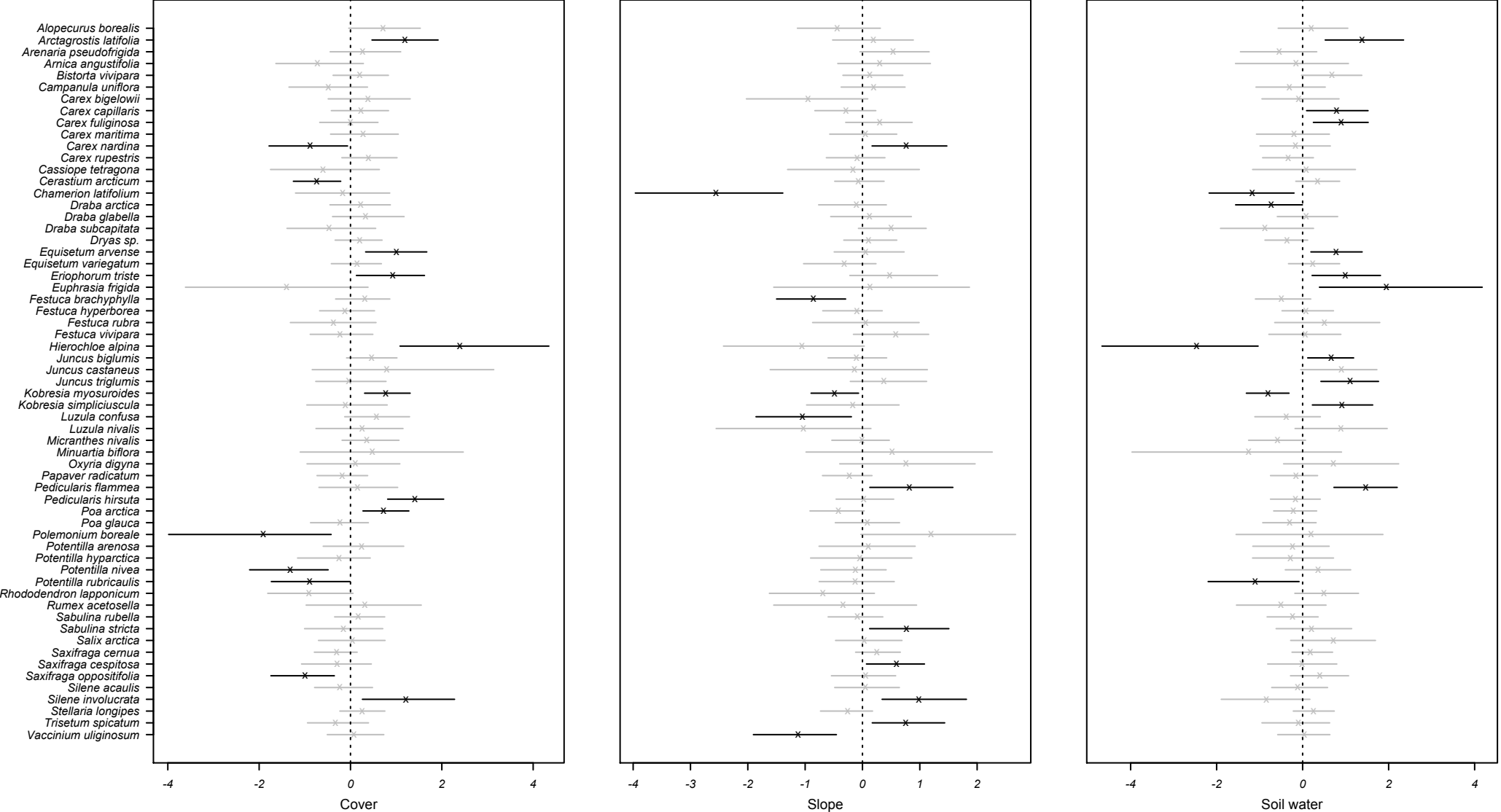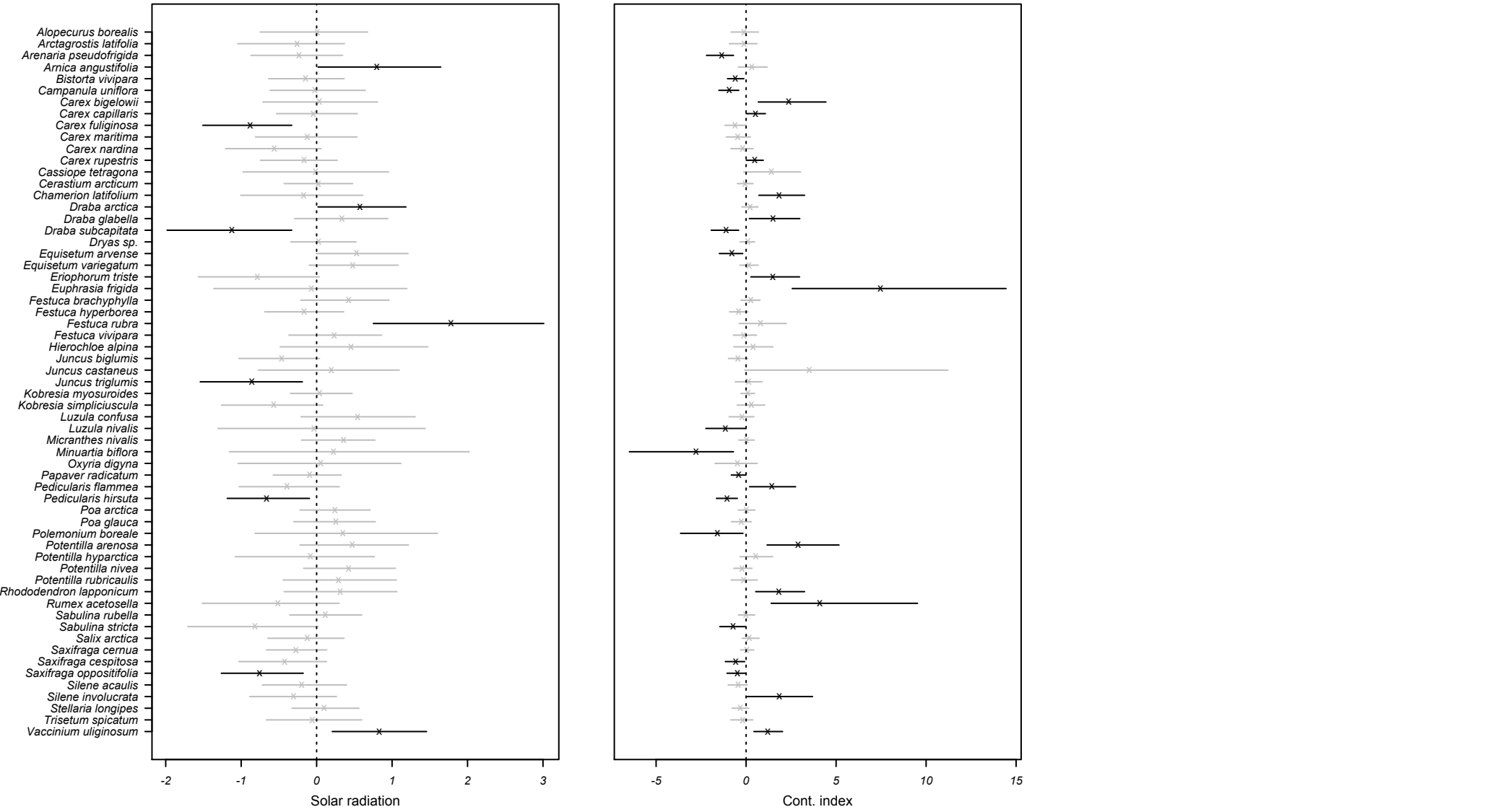

Supplement: Supplementary file 4 [file ECE3-7-10233-s004.pdf]
