# Supplementary material for: Plant community composition and species richness in the High Arctic tundra: From the present to the future
Source: Ecol Evol. 2017 Oct 25;7(23):10233–42. doi: 10.1002/ece3.3496 (PMC5723606; doi:10.1002/ece3.3496)
Supplement: Supplementary file 5 [file ECE3-7-10233-s005.docx]

**Plant community composition and species richness in the High Arctic tundra: from the present to the future – Supporting Information**

Jacob Nabe-Nielsen, Signe Normand, Francis K.C. Hui, Lærke Stewart, Christian Bay, Louise I. Nabe-Nielsen and Niels Martin Schmidt

**Taxonomic review of plant species in Young Sund.**

The review is based on the following online publications and databases:

### Annotated Checklist of the Panarctic Flora (PAF)

Elven, R., Murray, D.F., Razzhivin, V. & Yurtsev, B.A. (eds.) 2011. Annotated checklist of the Panarctic flora (PAF): vascular plants. Natural History Museum, University of Oslo (<http://nhm2.uio.no/paf/)>, accessed 4 October 2016.

### World Checklist of Selected Plant Families (WCSP)

*WCSP (2016). World checklist of selected plant families.* Facilitated by the Royal Botanic Gardens, Kew. Published on the Internet; <http://apps.kew.org/wcsp/>, accessed 4 October 2016.

### Flora of the Canadian Arctic Archipelago (FCAA)

Aiken, S.G., Dallwitz, M.J., Consaul, L.L., McJannet, C.L., Boles, R.L., Argus, G.W., Gillett, J.M., Scott, P.J., Elven, R., LeBlanc, M.C., Gillespie, L.J., Brysting, A.K., Solstad, H., and Harris, J.G. 2007. Flora of the Canadian Arctic Archipelago: descriptions, illustrations, identification, and information retrieval. NRC Research Press, National Research Council of Canada, Ottawa. <http://nature.ca/aaflora/data>, accessed 4 October 2016.

### Species 2000 & ITIS Catalogue of Life, 2016 Annual Checklist (CoL16)

Roskov Y., Abucay L., Orrell T., Nicolson D., Flann C., Bailly N., Kirk P., Bourgoin T., DeWalt R.E., Decock W., De Wever A., eds. (2016). Species 2000 & ITIS Catalogue of Life, 2016 Annual Checklist. Digital resource at [www.catalogueoflife.org/annual-checklist/2016](http://www.catalogueoflife.org/annual-checklist/2016). Species 2000: Naturalis, Leiden, the Netherlands. ISSN 2405-884X.

The data in current edition of the Catalogue of Life have been provided by 158 databases, including ITIS (see below) and WCSP. Data from this database are therefore not independent of ITIS and WCSP.

### Integrated Taxonomic Information System (ITIS)

Accessed 4 October 2016, from the Integrated Taxonomic Information System on-line database, [http://www.itis.gov](http://www.itis.gov/).

ITIS covers flowering plants from the US and Canada. It is based upon the cooperative work of John Kartesz, Biota of North America Program, North Carolina Botanical Garden, University of North Carolina at Chapel Hill, and the PLANTS database ([http://plants.usda.gov](http://plants.usda.gov/)) as of March 2000.

The data in these databases are not all fully updated and inconsistencies in the nomenclature may therefore reflect outdated data in some databases.

**Table S1.** List of plant species found in Young Sund using current nomenclature. The columns PAF (Panarctic Flora), WCSP (World Checklist of Selected Plant Families), FCAA (Flora of the Canadian Arctic Archipelago), CoL16 (Species 2000 & ITIS Catalogue of Life, 2016 Annual Checklist), and ITIS (Integrated Taxonomic Information System), indicate the support for the proposed scientific name in the different taxonomic databases included in the taxonomic review. The ‘Name in Böcher’ column gives the name used by Böcher et al. (1978).

| **Proposed name** | **Decision** | **Name in Böcher** | **PPAF** | **WWCSP** | **FFCAA** | **CoL16** | **IITIS** |
| --- | --- | --- | --- | --- | --- | --- | --- |
| *Alopecurus borealis* Trin. | New | *Alopecurus alpinus* Sm. | √ | ÷ | ÷ | ÷ | ÷ |
| *Arctagrostis latifolia* (R.Br.) Griseb. | = | *Arctagrostis latifolia* (R.Br.) Griseb. | √ | √ | √ | √ | √ |
| *Arenaria pseudofrigida* (Ostenf. & Dahl) Juz. ex Schischk. & Knorring | = | *Arenaria pseudofrigida* (Ostenf. & Dahl) Juz. | √ |  |  | √ | √ |
| *Armeria scabra* Pall. ex Roem. & Schult. | New | *Armeria scabra* ssp*. sibirica* (Turcz. ex Boiss.) Hyl. | √ |  | √ | √ | ÷ |
| *Arnica angustifolia* Vahl | = | *Arnica angustifolia* Vahl | √ |  | √ | √ | √ |
| *Betula nana* L. ssp. *nana* | New | *Betula nana* L. | √ | √ | √ | √ | √ |
| *Bistorta vivipara* (L.) Delarbre | New | *Polygonum viviparum* L. | √ |  | √ | √ | √ |
| *Braya glabella* ssp. *purpurascens* (R. Br.) Cody | New | *Braya purpurascens* (R. Br.) Bge. | √ |  | √ | √ | √ |
| *Braya humilis*  (C.A. Mey.) B.L. Rob. | = | *Braya humilis* (C.A. Mey.) B.L. Rob. | √ |  | √ | √ | √ |
| *Calamagrostis purpurascens* R.Br. | = | *Calamagrostis purpurascens* R.Br. | ssp. | √ | √ | √ | √ |
| *Campanula giesekiana* Vest ex Schult. | New | *Campanula gieseckiana* Vest, in Roem. & Schult. | ÷ | √ | ÷ | √ | √ |
| *Campanula uniflora* L. | = | *Campanula uniflora* L. | √ | √ | √ | √ | √ |
| *Cardamine bellidifolia*  L. | = | *Cardamine bellidifolia* L. | ssp. |  | √ | √ | √ |
| *Carex atrofusca* Schkuhr | = | *Carex atrofusca* Schkuhr | √ | √ | √ | √ | √ |
| *Carex bigelowii* Torr. ex Schwein. | = | *Carex bigelowii* Torr. ex Schwein. | √ | √ | ssp. | √ | √ |
| *Carex capillaris*  L. coll. | = | *Carex capillaris* L. coll. | √ | √ | ssp. | √ | √ |
| *Carex chordorrhiza* L.f. | = | *Carex chordorrhiza* L.f. | √ | √ | √ | √ | √ |
| *Carex fuliginosa* Schkuhr | New | *Carex misandra* R.Br. | √ | √ | ssp. | √ | √ |
| *Carex lachenalii* Schkuhr | = | *Carex lachenalii* Schkuhr | √ | √ | √ | √ | √ |
| *Carex marina* Dew. | = | *Carex marina* Dew. | √ | √ | √ | √ | √ |
| *Carex maritima* Gunn. | = | *Carex maritima* Gunn. | √ | √ | √ | √ | √ |
| *Carex nardina* Fr. | = | *Carex nardina* Fr. | √ | √ | √ | √ | √ |
| *Carex parallela* (Laest.) Sommerf. | = | *Carex parallela* (Laest.) Sommerf. | √ | √ |  | √ | √ |
| *Carex rupestris* All. | = | *Carex rupestris* All. | √ | √ | √ | √ | √ |
| *Carex saxatilis* L. | = | *Carex saxatilis* L. | √ | √ | √ | √ | √ |
| *Carex supina* ssp. *spaniocarpa* (Steud.) Hultén | = | *Carex supina* ssp. *spaniocarpa* (Steud.) Hultén | √ | var. | √ | var. | √ |
| *Cassiope tetragona* (L.) D. Don | = | *Cassiope tetragona* (L.) D. Don | ssp. |  | √ | √ | √ |
| *Cerastium alpinum* L. | New | *Cerastium alpinum* ssp. *lanatum* (Lam.) Ascherson & Graebner | √ |  | √ | √ | √ |
| *Cerastium arcticum* Lange | = | *Cerastium arcticum* Lange | √ |  | √ | √ | √ |
| *Chamerion latifolium* (L.) Holub | New | *Chamaenerion latifolium* (L.) Sweet | √ |  | √ | ÷ | √ |
| *Cochlearia groenlandica* L. | = | *Cochlearia groenlandica* L. | √ |  | √ | √ | √ |
| *Comastoma tenellum* (Rottb.) Toyok. | New | *Gentiana tenella* Rottb. | √ |  | √ | √ | ÷ |
| *Cystopteris fragilis* (L.) Bernh. | = | *Cystopteris fragilis* (L.) Bernh. | √ |  | √ | √ | √ |
| *Deschampsia brevifolia* R. Br. | = | *Deschampsia brevifolia* R. Br. | √ | ÷ | √ | ÷ | √ |
| *Draba arctica* J. Vahl | = | *Draba arctica* J. Vahl | √ |  | √ | √ | √ |
| *Draba fladnizensis* Wulfen | = | *Draba fladnizensis* Wulfen | √ |  | √ | √ | √ |
| *Draba glabella* Pursh | = | *Draba glabella* Pursh | √ |  | √ | √ | √ |
| *Draba lactea* Adams | = | *Draba lactea* Adams | √ |  | √ | √ | √ |
| *Draba nivalis* Lilj. | = | *Draba nivalis* Lilj. | √ |  | √ | √ | √ |
| *Draba oxycarpa* Sommerf. | New | *Draba gredinii* E. Ekman | √ |  |  | √ | √ |
| *Draba pauciflora* R. Br. | New | *Draba adamsii* Ledeb. | √ |  | √ | √ | √ |
| *Draba subcapitata* Simmons | = | *Draba subcapitata* Simmons | √ |  | √ | √ | √ |
| *Dryas* L. | = | *Dryas* L. | √ |  | √ | √ | √ |
| *Dupontia fisheri* R.Br. | New | *Dupontia psilosantha* Rupr. | √ | √ | √ | √ | √ |
| *Empetrum nigrum* L. | = | *Empetrum nigrum* L. | √ |  | √ | √ | √ |
| *Equisetum arvense* L. | = | *Equisetum arvense* L. | √ |  | √ | √ | √ |
| *Equisetum variegatum* Schleich. ex Weber & Mohr | = | *Equisetum variegatum* Schleich. | √ |  | √ | √ | √ |
| *Erigeron* L. | = | *Erigeron* L. | √ |  | √ | √ | √ |
| *Eriophorum callitrix* Cham. ex C.A. Mey. | = | *Eriophorum callitrix* Cham. | √ | √ | √ | √ | √ |
| *Eriophorum scheuchzeri* Hoppe | = | *Eriophorum scheuchzeri* Hoppe | √ | √ | √ | √ | √ |
| *Eriophorum triste* (Th. Fr.) Hadac & Á. Löve | = | *Eriophorum triste* (Th. Fr.) Hadac & Á. Löve | √ | ÷ | ÷ | ÷ | ÷ |
| *Euphrasia frigida* Pugsley | = | *Euphrasia frigida* Pugsley | √ |  |  | √ | √ |
| *Eutrema edwardsii* R. Br. | = | *Eutrema edwardsii* R. Br. | √ |  | √ | √ | √ |
| *Festuca brachyphylla* Schult. & Schult. f. | = | *Festuca brachyphylla* Schult. & Schult. f. | √ | √ | √ | √ | √ |
| *Festuca hyperborea* Holmen ex Fred. | = | *Festuca hyperborea* Holmen ex Fred. | √ | √ | √ | √ | √ |
| *Festuca rubra* L. ssp. *richardsonii* (Hooker) Hultén | New | *Festuca rubra* L. ssp. *arctica* (Hack.) Govor. | √ | sp. | √ | sp. | ÷ |
| *Festuca vivipara* (L.) Sm. s.l. | = | *Festuca vivipara* (L.) Sm. | √ | √ |  | √ | √ |
| *Hierochloë alpina* (Sw. ex Willd.) Roem. & Schult. | = | *Hierochloë alpina* (Willd.) Roem. & Schult. | √ | ÷ | √ | √ | ÷ |
| *Huperzia selago* (L.) Bernh. ex Schrank & Mart. | = | *Huperzia selago* (L.) Bernh. ex Schrank & Mart. | √ |  | √ | √ | √ |
| *Juncus biglumis* L. | = | *Juncus biglumis* L. | √ | √ | √ | √ | √ |
| *Juncus castaneus* Sm. | = | *Juncus castaneus* Sm. | ÷ | √ | √ | √ | √ |
| *Juncus triglumis* L. | = | *Juncus triglumis* L. | √ | √ | √ | √ | √ |
| *Kobresia myosuroides* (Vill.) Fiori | = | *Kobresia myosuroides* (Vill.) Fiori | √ | ÷ | √ | √ | √ |
| *Kobresia simpliciuscula* (Wahlenb.) Mack. | = | *Kobresia simpliciuscula* (Wahlenb.) Mack. | √ | ÷ | √ | √ | √ |
| *Koenigia islandica* L. | = | *Koenigia islandica* L. | √ | √ | √ | √ | √ |
| *Luzula confusa* Lindeb. | = | *Luzula confusa* Lindeb. | √ | √ | √ | √ | √ |
| *Luzula nivalis* (Laest.) Spreng. | New | *Luzula arctica* Blytt | √ | √ | √ | √ | √ |
| *Luzula wahlenbergii* Rupr. | = | *Luzula wahlenbergii* Rupr. | √ | √ | √ | √ | √ |
| *Micranthes foliolosa* (R. Br.) Gornall | New | *Saxifraga foliolosa* R. Br | √ |  | √ | √ | √ |
| *Micranthes nivalis* (L.) Small | New | *Saxifraga nivalis* L. | √ |  | √ | √ | √ |
| *Micranthes tenuis* (Wahlenb.) Small | New | *Saxifraga tenuis* (Wahlenb.) Harry Sm. | √ |  | √ | √ | √ |
| *Minuartia biflora* (L.) Schinz & Thell. | = | *Minuartia biflora* (L.) Schinz & Thell. | √ |  | √ | ÷ | √ |
| *Oxyria digyna* (L.) Hill | = | *Oxyria digyna* (L.) Hill | √ |  | √ | √ | √ |
| *Papaver radicatum* Rottb. coll. | = | *Papaver radicatum* Rottb. coll. | √ |  | √ | √ | √ |
| *Pedicularis flammea* L. | = | *Pedicularis flammea* L. | √ |  | √ | √ | √ |
| *Pedicularis hirsuta* L. | = | *Pedicularis hirsuta* L. | √ |  | √ | √ | √ |
| *Pedicularis lapponica* L. | = | *Pedicularis lapponica* L. | √ |  | √ | √ | √ |
| *Physaria arctica* (Wormsk. ex Hornem.) O'Kane & Al-Shehbaz | New | *Lesquerella arctica* (Wormsk. ex Hornem.) S. Watson | √ |  | ÷ | √ | √ |
| *Poa abbreviata* R. Br. | = | *Poa abbreviata* R. Br. | √ | √ | √ | √ | √ |
| *Poa alpina* L. | = | *Poa alpina* L. | √ | √ | √ | √ | √ |
| *Poa arctica* R. Br. s.l. | = | *Poa arctica* R. Br. | √ | √ | ssp. | √ | √ |
| *Poa glauca* Vahl | = | *Poa glauca* Vahl | √ | √ | √ | √ | √ |
| *Poa pratensis* L. s.l. | = | *Poa pratensis* L. coll. | √ | √ | ssp. | √ | √ |
| *Polemonium boreale* Adams | = | *Polemonium boreale* Adams | √ | √ | √ | √ | √ |
| *Potentilla arenosa* (Turcz.) Juz. | New | *Potentilla hookeriana* Lehm. | √ |  | √ | √ | √ |
| *Potentilla hyparctica* Malte | = | *Potentilla hyparctica* Malte | √ |  | √ | √ | √ |
| *Potentilla nivea* L. | = | *Potentilla nivea* L. | √ |  | √ | √ | √ |
| *Potentilla rubricaulis* Lehm. | = | *Potentilla rubricaulis* Lehm. | ÷ |  | √ | √ | √ |
| *Ranunculus arcticus* Richardson | New | *Ranunculus affinis* R. Br. | √ |  | √ | ÷ | ÷ |
| *Ranunculus glacialis* L. | = | *Ranunculus glacialis* L. | √ |  |  | √ | √ |
| *Ranunculus hyperboreus* Rottb. | = | *Ranunculus hyperboreus* Rottb. | √ |  | √ | √ | √ |
| *Ranunculus nivalis* L. | = | *Ranunculus nivalis* L. | √ |  | √ | √ | √ |
| *Ranunculus pygmaeus* Wahlenb. | = | *Ranunculus pygmaeus* Wahlenb. | √ |  | √ | √ | √ |
| *Ranunculus sulphureus* Sol. | = | *Ranunculus sulphureus* Sol. | var. |  | √ | √ | √ |
| *Rhododendron lapponicum* (L.) Wahlenb. | = | *Rhododendron lapponicum* (L.) Wahlenb. | √ |  | √ | √ | √ |
| *Rumex acetosella* L. | = | *Rumex acetosella* L. | √ |  |  | √ | √ |
| *Sabulina rubella* (Wahlenb.) Dillenb. & Kadereit | New | *Minuartia rubella* (Wahlenb.) Hiern |  |  |  | √ |  |
| *Sabulina stricta* (Sw.) Rchb. | New | *Minuartia stricta* (Sw.) Hiern |  |  |  | √ | ÷ |
| *Salix arctica* Pall. | = | *Salix arctica* Pall. | √ |  | √ | √ | √ |
| *Saxifraga aizoides* L. | = | *Saxifraga aizoides* L. | √ |  | √ | √ | √ |
| *Saxifraga cernua* L. | = | *Saxifraga cernua* L. | √ |  | √ | √ | √ |
| *Saxifraga cespitosa* L. | New | *Saxifraga caespitosa* L. | √ |  | √ | √ | √ |
| *Saxifraga hirculus* L. | = | *Saxifraga hirculus* L. | √ |  | √ | √ | √ |
| *Saxifraga oppositifolia* L. | = | *Saxifraga oppositifolia* L. | √ |  | √ | √ | √ |
| *Saxifraga platysepala* (Trautv.) Tolm. | = | *Saxifraga platysepala* Trautv.) Tolm. | √ |  | √ | √ | ÷ |
| *Silene acaulis* (L.) Jacq. | = | *Silene acaulis* (L.) Jacq. | √ |  | √ | √ | √ |
| *Silene involucrata* (Cham. & Schltdl.) Bocquet | New | *Melandrium affine* J. Vahl coll. | √ |  | √ | √ | √ |
| *Silene sorensenis* (B. Boivin) Bocquet | New | *Melandrium triflorum* (R. Br.) J.Vahl | √ |  | √ | √ | √ |
| *Silene uralensis* ssp. *arctica* (Th. Fr.) Bocquet | New | *Melandrium apetalum* (L.) Fenzl ssp. *arcticum* (Th.Fr.) Hultén | √ |  | √ | ÷ | ÷ |
| *Stellaria longipes* Goldie | = | *Stellaria longipes* Goldie | √ |  | √ | √ | √ |
| *Taraxacum arcticum* (Trautv.) Dahlst. aggregate | = | *Taraxacum arcticum* (Trautv.) Dahlst. aggregate | √ |  | √ | √ | ÷ |
| *Tofieldia coccinea* Richardson | = | *Tofieldia coccinea* Richardson | √ | √ | √ | √ | √ |
| *Tofieldia pusilla* (Michx.) Pers. | = | *Tofieldia pusilla* (Michx.) Pers. | √ | √ | √ | √ | √ |
| *Trisetum spicatum* (L.) K. Richt. | = | *Trisetum spicatum* (L.) K. Richt. | √ | √ | √ | √ | √ |
| *Vaccinium uliginosum* L. | = | *Vaccinium uliginosum* L. | √ |  | ssp. | √ | √ |
| *Woodsia glabella* R. Br. ex Richardson | = | *Woodsia glabella* R. Br. ex Richardson | √ |  | √ | √ | √ |

### Legend

| Proposed name accepted | √ | Proposed name accepted, but species found in Young Sund demoted to subspecies | ssp. |
| --- | --- | --- | --- |
| Proposed name not accepted | ÷ | Proposed name accepted, but species found in Young Sund demoted to a variety (var.) | var. |
| Proposed name not included |  | Proposed name accepted, but subspecies name used for Young Sund taxon promoted to species level | sp. |

### Taxonomic updates

In the following we provide a brief description of the rationale behind choosing a new name in the cases where the name proposed by Böcher et al. (1978) was not accepted. In the cases where we decided to retain the name used by Böcher et al. in spite of differences of opinion among the studied databases we also present rationale for doing so. Each of the names used by Böcher et al. corresponds to one new name (no splitting of taxa), and each new name corresponds to only one species name in Böcher et al. (no lumping of taxa). The only exception is *Dupontia psilosanta* that was merged with *Dupontia fisheri* R.Br. This one-to-one relationship does not apply outside the Young Sund region for all species.

### Alopecurus borealis Trin.

The name *Alopecurus alpinus* Sm. (1803) is illegitimate, as it is a later homonym of the previously described non-arctic species *Alopecurus alpinus* Vill. (1786) from the western Alps (FCAA, PAF, WCSP, CoL16). Instead the name *Alopecurus magellanicus* Lam. is accepted for *Alopecurus alpinus* Sm. in all databases except the PAF, following Soreng *et al.* (2003).

The *Alopecurus magellanicus* aggregate proposed by Soreng *et al.* is, however, present in both northern-alpine and arctic areas and in southern South America, separated by a wide gap across the tropical and subtropical belts. As noted by Elven and Murray in PAF the revision by Soreng *et al.* was focused on southern hemisphere plants. There is considerable morphological variation between plants from different parts of the range, and molecular or morphological analyses that could support a merger of the southern and northern hemisphere taxa appear to be lacking. Until such evidence is provided we tentatively accept the name *Alopecurus borealis* Trin. for the circumpolar and alpine part of the range, following Elven and Murray. The plants on Greenland belong to this group.

### Armeria scabra Pall. ex Roem. & Schult.

We accept *Armeria scabra* for the species named *Armeria scabra* ssp. s*ibirica* by Böcher et al. (1978)*,* following all databases where the species was mentioned. FCAA use the study of Lefèbvre & Vekemans (1994) to support this decision. This study used phenetic analysis based on several hundred herbarium specimens of *Armeria maritima* to identify two subspecies occupying distinct regions: *A. maritima* subsp. *californica*, occurring under temperate climates along the Pacific Coast of the USA, from Vancouver Island to California, and *A. maritima* subsp. *sibirica*, [= *A. scabra*] found in arctic and subarctic areas. PAF find no reason to treat the subspecies as anything but two species: *A. maritima* and *A. scabra*.

### Betula nana L. ssp. nana

PAF, FCAA and ITIS accept *Betula nana* subsp*. nana* as the subspecies occurring in both eastern and western Greenland. PAF notes that: “Two major races of *Betula nana* have been accepted for a long time: the very broadly amphi-Atlantic subsp. *nana* and the similarly broadly amphi-Beringian subsp. *exilis*. These two races are fairly well separated morphologically and find support in molecular data (Eidesen 2007, AFLP).”

### Bistorta vivipara (L.) Delarbre

This species, which was named *Polygonum viviparum* by Böcher et al. (1978), is treated under the name *Bistorta* *vivipara* in all the checked databases (except WCSP), but the reason for doing so is not provided. In the Plant List ([http://www.theplantlist.org/tpl1.1/record/kew-2572250, accessed 7 October 2016)](http://www.theplantlist.org/tpl1.1/record/kew-2572250,%20accessed%207%20October%202016)), *Persicaria* *vivipara* (L.) Ronse Decr is the accepted name for *Polygonum viviparum* L. and *Bistorta* *vivipara* (L.) Delarbre is a synonym. The record derives from [WCSP (in review)](http://www.theplantlist.org/1.1/about/#wcsir) (data supplied 23 March 2012), which reports *Persicaria* *vivipara* as an [accepted](http://www.theplantlist.org/1.1/about/#accepted) name. In spite of this we accept *Bistorta vivipara* (L.) Delarbre since it is currently the most widely accepted name.

### Braya glabella ssp. purpurascens (R. Br.) Cody

We accept the name *Braya glabella* ssp. *purpurascens,* which was called *Braya purpurascens* by Böcher et al. As mentioned in PAF, Harris (1985) synonymized *Braya purpurascens*, *B. henryae*, *B. bartlettiana*, and some other taxa with *B. glabella*. This view was later adopted by Rollins (1993) and found support in the molecular data and the phylogeny presented by Warwick et al. (2003). According to Elven and Murray in PAF “The proposed taxa of *B. glabella* s.l. have rather distinctive geographical patterns. The most widespread one is the high-arctic circumpolar *B. purpurascens*, the next most widespread one is the low-arctic and boreal North American *B. glabella* s.s., whereas three others are fairly local […]. A late divergence could explain the lack of differentiation in the gene sequences analysed yet. This lack of genetic differentiation also supports the recognition of subspecies (i.e., regional races) rather than species”. For these reasons PAF accepts Cody’s (1994) recombination of *B. purpurascens* as a subspecies. This was done also by Harris (2010) for Flora of North America.

### Calamagrostis purpurascens R.Br.

The name *Calamagrostis purpurascens* R.Br. was adopted by all databases except PAF, where the name *Calamagrostis purpurascens* subsp. *purpurascens* is preferred for the taxon occurring in eastern Greenland. The distinction between the different subspecies is based on minor morphological characters between plants in North America and those in eastern Canada and Greenland (cf. Elven and Murray, in PAF).

### Campanula [giesekiana Vest ex Schult.](#_Campanula_rotundifolia_L._2)

We accept the name *Campanula giesekiana* L. for the taxon named *C. gieseckiana* by Böcher et al. (1978), following WCSP, CoL16 and ITIS. See Shetler (1982) regarding the orthography, omitting the c in 'giese(c)kiana'. Shelter (1982) argues that the nearctic harebells should all be considered several variable ecotype 'races' of *Campanula rotundifolia* L., PAF considers them as subspecies of *Campanula rotundifolia* L., whereas FCAA accepts the species *Campanula rotundifolia* L. for harebells in the Canadian Arctic Archipelago. However Lammers (2007) restricts *C. rotundifolia* to the "Europe to S. Siberia, Manchuria [...], & Sakhalin" and recognizes a number of North American taxa separately. ITIS, WCSP and CoL16 follow Lammers in this, pending further evaluation.

### Cardamine bellidifolia L.

The name *Cardamine bellidifolia* L. was adopted by all databases, except PAF where the name *Cardamine bellidifolia* subsp. *bellidifolia* L. was preferred. We have, however, not found any support for dividing the species into subspecies.

### Carex bigelowii Torr. ex Schwein.

The name Carex bigelowii *Torr.* ex Schwein was adopted by all databases, except FCAA where the name *Carex bigelowii* ssp. *bigelowii* was preferred. In PAFF Elven and Schönswetter also accept the rank of subspecies following results from molecular investigations but without taking the nomenclatural consequences. They also note that “*Carex bigelowii* s.l. is a polymorphic and widespread arctic-alpine complex” and “If only one collective species is considered, the priority name is *C. bigelowii* Torr.”.

### Carex capillaris L. coll.

The name *Carex capillaris* L. coll. was adopted by all databases, except PAFF. In PAFF three northern races of *Carex capillaris* are recognised: Ssp. *capillaris* (temperate-boreal European type), ssp. *fuscidula* (main arctic race in both North America and Eurasia) and var. *elongate* (boreal North American plants). FCAA, WCSP and CoL16 also recognise races of *Carex capillaris,* but ITIS does not. We prefer to maintain the collective treatment of *Carex capillaris* L. coll. which was also used by Böcher et al. (1978).

### Carex fuliginosa Schkuhr

We use the name *Carex fuliginosa* Schkuhr for the species called *Carex misandra* by Böcher et al. (1978). This name is adopted by all databases, except FCAA where the name *Carex fuliginosa* ssp. *misandra* is used. There is some disagreement among the contributors to PAF as to whether subspecies can be recognised, but until further studied we follow Ball in not recognising different subspecies.

### Carex supina ssp. spaniocarpa (Steud.) Hultén

WCSP and CoL16 use the name *Carex supina* var. *spaniocarpa* instead of *C. supina* ssp. *spaniocarpa*. However we find no support for changing the status of *Carex supina* ssp. *spaniocarpa*.

### Cassiope tetragona (L.) D. Don

In PAF the subspecies name *Cassiope tetragona* subsp. *tetragona* (L.) D. Don. is the only accepted name for *Cassiope tetragona*. However we have not found any support for dividing the species into subspecies.

### Cerastium alpinum L.

We accept the name *Cerastium alpinum* (omitting ssp. *lanatum* used by Böcher) based on the arguments presented in FCAA: *“Cerastium alpinum* from Greenland and other Arctic areas has been referred to subsp. *lanatum* by several authors (e.g., Hultén 1956). This subspecies was, however, described from central Europe and has a relatively thermophilous, montane to low alpine distribution in central Europe and Fennoscandia. The arctic representatives differ from the central and northern European subsp. *lanatum* in several morphological characters (they are generally coarser, have long, narrow, and more acute leaves, larger flowers and fruits, and a coarser indumentum). The lanate plants of Arctic areas have probably evolved independently of the thermophilous plant of central and northern Europe, and the subspecific name should be avoided for arctic populations”. This view is also presented by Elven and Elvebakk (1996).

### Chamerion latifolium (L.) Holub

The name *Chamerion latifolium* (L.) Holub was adopted for the species named *Chamaenerion latifolium* by Böcher et al. (1978), in accordance with PAF, FCAA and ITIS. Holub (1972) presents a clear explanation of the choice of *Chamerion* as a genus name instead of *Chamaenerion*. CoL16 treats the name *Chamerion latifolium* (L.) as a synonym for *Epilobium latifolium* L.

### Comastoma tenellum (Rottb.) Toyok.

PAF, FCAA and CoL16 accept *Comastoma* *tenellum* (Rottb.) Toyok. to replace *Gentiana tenella*, but they do not explain why. ITIS accepts *Gentianella tenella* ssp. *tenella* [(Rottb.) Börner](https://www.itis.gov/servlet/SingleRpt/RefRpt?search_type=author&search_id=author_id&search_id_value=145016). We choose to follow the majority of the databases and accept the name *Comastoma* *tenellum*.

### Deschampsia brevifolia R. Br.

We maintain the name *Deschampsia brevifolia* R. Br. as used by Böcher et al. (1978), following PAF, FCAA and ITIS. WCSP and CoL16 use the name *Deschampsia cespitosa* ssp. *cespitosa* and treat the name *Deschampsia brevifolia* R. Br. as a synonym. The northern representatives of *Deschampsia* are closely related and have all sometimes been considered races of one species: *D. cespitosa* (e.g. Tzvelev 1976). According to Aiken, Elven, Murray, and Tzvelev (in PAF) there is, however, little evidence for intergradation of the different Eurasian and North American subspecies of *C. cespitosa* s.l. The co-occurrence of two or more morphologically separable species within a region is the rule rather than the exception (in northern Europe *D. cespitosa* s.s. and *D. alpina*; in the High Arctic *D. sukatschewii* and *D. brevifolia*; in the North Atlantic areas also *D. alpina*). For these reasons we concur with PAF and accept these taxa as species.

### Draba oxycarpa Sommerf.

We tentatively accept the name *Draba oxycarpa* for the species called *Draba gredinii* by Böcher et al. (1978), following PAF and CoL16. We do so based on the arguments presented in PAF: *“Draba oxycarpa* was described from Svalbard and the Norwegian mainland, based on collections by B.M. Keilhau (Sommerfelt 1833). The name *D. oxycarpa* Sommerf. 1833 predates *D. oxycarpa* Boiss. 1849 (from western Asia), is validly published, and can be substatiated by a type […]. If confirmed, *D. oxycarpa* will be the earliest name for the arctic, octoploid species later described by (Ekman 1933) as *D. gredinii*.”

### Draba pauciflora R. Br.

We accept the name *Draba pauciflora* for the species named *Draba adamsii* by Böcher et al. (1978). According to PAF, FCAA, CoL16, ITIS *D. pauciflora* is a synonym for *D. adamsii*.

### Dupontia fisheri R.Br. s.l.

We include *Dupontia psilosantha* Rupr. in *Dupontia fisheri* R.Br. based on the arguments presented in PAF: “Brysting et al. (2003) studied *Dupontia* by numerical taxonomy from herbarium specimens, including types and chromosome vouchers. They concluded that morphological characters used in the literature to divide the genus into more than one taxon cannot be reliably applied to distinguish most North American plants. In North America, we might see two endpoints, which correspond more or less to *D. psilosantha* and *D. fisheri*, respectively […]. However, as suggested by Polunin (1940), there appears to be a continuum of variation among North American specimens and too many intermediate forms exist, which are impossible to place in two or three categories […]. Until further evidence is available, Brysting et al. suggest that the genus is treated as monotypic […].

Molecular evidence supports the use of a wide species circumscription (Brysting *et al.* 2004). DNA sequence data (trnL-trnF, ITS; including plants from Russia, Svalbard, Canada, and Alaska) revealed almost no variation within *Dupontia*, only a couple of base pair changes, which were related neither to ploidy levels, nor to geography.”

### Eriophorum triste (Th. Fr.) Hadac & Á. Löve

All the searched databases except PAF treat *Eriophorum triste* (Th. Fr.) Hadac & Á. Löve as a synonym for *Eriophorum angustifolium* ssp. *triste* (Th. Fr.) Hultén. Elven and Murray (in PFA), however, accept *E. triste* as a distinct and widespread species in the northern and alpine parts of North America and in Greenland. They argue that “The separating features from *E. angustifolium* are not restricted to the scabrous peduncles (which North American authors have looked at and misinterpreted, see *E. komarovii*), but the subrotund and always short-pedunculate spikes, the white to slightly greyish wool, the lowermost subtending bract which is dark grey to black to the margins, and the flat and spreading basal leaves. These characters immediately distinguish *E. triste* both from *E. angustifolium* and from the scabrous-peduncled *E. komarovii* (spikes narrowly oblong or ovoid, wool often pinkish, lowermost subtending bract hyaline-margined and reddish, peduncles extended, and basal leaves nearly filiform and erect)”. As there are not convincing arguments for replacing *Eriophorum triste* with *Eriophorum angustifolium* ssp. *triste*we maintain the name *Eriophorum triste,* following PAF.

### Festuca rubra subsp. richardsonii (Hook.) Hultén

According to PAF *Festuca rubra* and its close relatives are an unusually complicated group. PAF recognises *Festuca rubra* subsp. *richardsonii* (Hook.) Hultén as the major race throughout the Arctic and notes that it is morphologically fairly uniform throughout its range. PAF notes that “subspecies *richardsonii* differs from subsp. *rubra* in several, assumed independently inherited characters”.

PAF notes that the correct name for *Festuca rubra* subsp. *richardsonii* as subspecies is undecided and that the majority of authors apply subsp. *arctica* (Hack.) Govor. based on Hackel's f. *arctica* (Soreng 2003; Darbyshire & Pavlick 2007)”. Böcher et al. (1978) also apply *Festuca rubra* ssp*. arctica*. PAF and FCAA however argue that no exact reference to a type of Hackel's *'arctica'* had been found whereas there is a clearly identifiable type of Hooker's *'richardsonii'* .PAF and FCAA have therefore chosen to apply ssp. *richardsonii* until a type for Hackel's *"arctica"* can be confirmed. ITIS is the only one of the searched database that applies subsp. *arctica.*

According to PAF *Festuca rubra* subsp. *richardsonii* may optionally be promoted to species, “although there are frequent transitions between subsp. *richardsonii* and subsp. *rubra*, at least in northwestern Europe and Greenland.” CoL16 and WCSP already accept *Festuca richardsonii* at species level.

### Festuca vivipara (L.) Sm. s.l.

Elven, Aiken, and Tzvelev recognises in PAF three viviparous species in Greenland: *Festuca frederikseniae* E.B. Alexeev (syn.: *Festuca vivipara* var. *hirsuta* Schol.), *Festuca vivipara* (L.) Sm. and *Festuca viviparoidea* Krajina ex Pavlick (syn.: *F. vivipara* L. subsp. *glabra* Frederiksen). They agree that they are “separable and non-overlapping morphologically, that they probably are viviparous derivatives among sexual species or more probably their hybrids, and that they have attained morphological consistency and independent geographical ranges that justify their acceptance.” The three species are also accepted by WCSP, CoL16 and ITIS.

We choose a collective treatment of *Festuca vivipara* since that’s how it is treated in Böcher (1978) – the flora used to determine the specimens. We therefore don’t know whether the registered plants belong to *Festuca vivipara* s.s. or *F. vivipara* L. subsp. *glabra* (*Festuca viviparoidea*)*.* It is unlikely that any of our specimens are *Festuca frederikseniae* since it is treated in Böcher as a southern (i.e. not in Young Sound) variation with densely hairy spikelets - *Festuca vivipara* var. *hirsuta.*

### Hierochloë alpina (Sw. ex Willd.) Roem. & Schult.

We maintain the name *Hierochloë alpina* as used by Böcher et al. (1978) and as suggested by PAF, FCAA and CoL16. WCSP and ITIS include *Hierochloë* in the genus *Anthoxanthum* and accept the name *Anthoxanthum monticola* (Bigelow) Veldkamp instead of *H. alpina.* In PAF the argument for maintaining *Hierochloë* as a distinct genus is that the two genera are morphologically rather different in Europe, northern Asia and North America, and that Schouten and Veldkamp (1985) proposed merging the two genera based on morphological similarities for Asian representatives. Soreng et al. (2003) and Allred and Barkworth (2007) accepted the merger for America. For the northern representatives there is a difference in chromosome number for the two genera, with n=5 in *Anthoxanthum* s.s. and n=7 in *Hierochloë*, which counts against merging the two genera.

### Juncus castaneus Sm.

We maintain the name *Juncus castaneus* as used by Böcher et al. (1978) and suggested by WCSP, FCAA, ITIS and CoL16. In PAF Elven, Murray and Solstad accepted two species for the checklist: *J. castaneus* and *J. leucochlamys*. They found the inspected specimens from northeastern Asia, North America, Greenland, and Svalbard to differ in some consistent features from the material from northern mainland Europe and northwestern Siberia. They proposed using the name *J. leucochlamys* for the former and *J. castaneus* for the latter. For Alaska, Hultén (1943) first accepted two species, but later changed his opinion because the taxa seemed to intergrade morphologically Hultén (1968). Brooks and Clemants (2000) synonymized *J. leucochlamys* with *J. castaneus* for North America. Due to divergence in opinions among different studies based on plant morphology, we are reluctant to replace the name *J. castaneus* with *J. leucochlamys* until there is genetic evidence to support it.

### Kobresia myosuroides (Vill.) Fiori, and Kobresia simpliciuscula (Wahlenb.) Mack.

All the searched databases except WCSP accept the names *Kobresia myosuroides* and *Kobresia simpliciuscula.* WCSP does not accept *Kobresia* as a genus apart from *Carex* and suggest the names *Carex simpliciuscula* Wahlenb. and *Carex myosuroides* Vill. In PAF it is noted that “Some molecular evidence throws doubts on whether it is possible to keep *Kobresia* (and some other genera) consistently apart from *Carex* (e.g., Yen and Olmstead 2000)”. As most databases maintain the genus *Kobresia* we maintain this name, pending further evaluation.

### Luzula nivalis (Laest.) Spreng.

We accept the name *Luzula nivalis* for the species named *Luzula arctica* in Böcher et al. (1978). All the searched databases replace *Luzula arctica* Blytt with *Luzula nivalis* (Laest.) Spreng.

Egorova et al. (in PAF) argue that “The application of *L. arctica* is based on arguments by Hylander (1945, 1953) that the earlier name *L. nivalis* Sprengel 1825 refers to another plant. However, Sprengel's name is explicitly based on *L. campestris* var. *nivalis* Laest., and the application must follow the type of the basionym. The lectotypes of Blytt's *"arctica"* (from Norway) and Laestadius' *"nivalis"* (from Sweden) belong to the same taxon. Kirschner’s (2001) designation of a lectotype of *L. campestris* var. *nivalis* finishes this issue.”

### Micranthes foliolosa (R. Br.) Gornall, Micranthes nivalis (L.) Small and Micranthes tenuis (Wahlenb.) Small

*Saxifraga* s.l. has morphologically distinct sections that have repeatedly been considered as different genera (Zhmylev, in PAF). We follow Zhmylev and the other searched databases and accept the genus *Micranthes* for the species named *Saxifraga foliolosa, Saxifraga nivalis* and *Saxifraga tenuis* in Böcher (1978).

Elven and Murray note in PAF that: “The phylogenetic analyses of Soltis et al. (1996, 2001) and later works show that there are two major branches of genera in Saxifragaceae. A major segregate of traditional *Saxifraga*, i.e., the Micranthes group together with, e.g., *Chrysosplenium*, belongs in a different main branch from that of *Saxifraga* s.s. The molecular evidence makes it impossible to retain *Micranthes* within *Saxifraga* and we accept a genus as done by Brouillet and Gornall (2007) and Brouillet and Elvander (2009).”

### Minuartia biflora (L.) Schinz & Thell., Sabulina rubella (Wahlenb.) Dillenb. & Kadereit and Sabulina stricta (Sw.) Rchb.

Recent molecular studies by Dillenberger and Kadereit (2014) have determined *Minuartia* to be polyphyletic. The clade to which *Minuartia stricta* and *Minuartia rubella* belong has been segregated as a distinct genus, *Sabulina* Rchb., whereas the clade to which *Minuartia biflora* belongs has been segregated to a generic rank were the earliest available name is *Cherleria* L. (Dillenberger & Kadereit 2014).

CoL16 accepts the new circumscription of *Minuartia*, whereas the other four searched databases have not yet dealt with the studies by Dillenberger and Kadereit (2014) and maintain the name *Minuartia* in its collective meaning. We follow Dillenberger and Kadereit (2014) and accept the name changes proposed in CoL16 for *Minuartia stricta* and *Minuartia rubella.* CoL16 only provisionally accepts the name *Cherleria biflora* (L.) comb. ined. to replace *Minuartia biflora*. We therefore keep *Minuartia biflora* in *Minuartia* until a revision of *Cherleria* has been published.

### Physaria arctica (Wormsk. ex Hornem.) O'Kane & Al-Shehbaz

We accept the name *Physaria arctica* for the species named *Lesquerella arctica* in Böcher et al. (1978), following PAF, CoL16 and ITIS. These databases accept to transfer the genus *Lesquerella* S. Watson to *Physaria* A. Gray. Murray and Elven (in PAF) note that Al-Shehbaz and O'Kane (2002) presented molecular evidence suggesting that *Lesquerella* should be merged with the southwestern North American genus *Physaria* under the latter priority name. The case was further argued for by O'Kane (2010). For this reason PAF accepts *Physaria* in preference of *Lesquerella* for the two species that reach the Arctic.

This change was not accepted by FCAA, that argue that “This species was transferred to the genus *Physaria*, a small genus of plants endemic to an area near Colorado, by O'Kane and Al-Shehbaz (2002), based on DNA evidence. Mulligan (personal communication, 2005) indicated that plant morphology and the morphology of the chromosomes do not support this transfer [Novon 12: 319–329. 2002], and this is to be followed in the *Flora of North America* treatment (Ihsan Al-Shehbaz , personal communication, Dec. 2003).” However O’Kane (2010) uses the name *Physaria arctica* in the Flora of North America rather than *Lesquerella arctica.*

We have not seen any resent arguments for the FCAA treatment of the genus and therefore accept the name *Physaria arctica* replacing *Lesquerella arctica.*

### Poa arctica R. Br. s.l.

We maintain the collective treatment of *Poa arctica* used by Böcher et al. (1978). PAF notes that *Poa arctica* s.l. is a large and much disputed group of northern *Poa*s. Elven, Alsos and Haugen (in PAF) recognise two disjunctly different groups ("arctica" and "caespitans") without signs of transition in Greenland and northern Europe, suggesting that var. *arctica* and var. *caespitans* can be recognised. FCAA recognises the two groups as subspecies (ssp. *arctica* and ssp. *caespitans*) and does not use a collective treatment of *Poa arctica*.

### Poa pratensis L. s.l.

We maintain the collective treatment of *Poa pratensis* used by Böcher et al. (1978) due to difficulties distinguishing different groups within the taxon. PAF notes that “*Poa pratensis* s.l. is another partly or mainly agamic complex (many studies from Müntzing (1932, 1940 and onwards) treated in several ways in different traditions. The limits between the taxa (agamospecies) are unclear and some reticulation is probable.” The contributors agree that there may be four or five widespread main groups in the northern regions known under the names *P. pratensis* s.s., *P. angustifolia*, *P. alpigena*, *P. irrigata* (= *P. humilis* = *P. subcaerulea*), and perhaps *P. colpodea*.

### Potentilla arenosa (Turcz.) Juz.

We accept the name *Potentilla arenosa* for the species named *Potentilla hookeriana* in Böcher et al. (1978), in agreement with all the searched databases. In FCAA the replacement of names is reasoned as follows: “The name *P. arenosa* is accepted over *P. hookeriana* after a reconsideration of the types (Elven, personal communication, 2005). Jurtsev and Soják (personal communication) have long asserted that Lehmann's type of *P. hookeriana*, which has a restricted distribution in the southern Canadian Cordillera, is possibly a hybrid, and is inapplicable for the widespread arctic-boreal plant(s). Bente Eriksen (personal communication) has also studied the type and concurs. This means that the priority name must be *P. arenosa*, if we consider the Russian and the American plants as the same, which we do.”

### Potentilla rubricaulis Lehm.

We maintain the name *Potentilla rubricaulis* that was also used by Böcher et al. (1978), following FCAA, CoL16 and ITIS. In PAF Elven and Murray argue that plants assigned as *P. rubricaulis* from the Canadian Arctic Archipelago and Greenland belong to three different taxa: “one yet unnamed for which they provisionally apply the name *P.* sp. aff. *uschakovii*, one they accept as *P. pedersenii*, and one yet unnamed for which they provisionally apply the name *P.* sp. aff. *vahliana*. *Potentilla pedersenii* Rydb. appears to be the name used for plants identified as *Potentilla rubricaulis* in Greenland. FCAA has chosen a collective treatment of the aggregate and notes that the *P. rubricaulis* aggregate may be an artificial group of agamospermic plants derived from hybrids between several species of sect. *Niveae* (both the *P. nivea* group and the *P. uniflora* group) and at least two species of sect. *Multifidae* (*P. anachoretica*, *P. pulchella*, perhaps also *P. bimundorum* in the case of *P. rubricaulis* s.s.). Some of the entities in the aggregate are widespread, but vary morphologically from site to site. They choose a collective treatment of the group due to lack of the data needed for a detailed taxonomic treatment.

### Ranunculus arcticus Richardson

We accept the name *Ranunculus arcticus* for the species named *Ranunculus affinis* in Böcher et al. (1978), following PAF and FCAA*.* Rebristaya and Elven (in PAF) note that Richardson's name *Ranunculus arcticus* from March 1823 predates Robert Brown's *R. affinis* from late 1823, and assume that the names refer to the same species. They argue that the name *R. pedatifidus* Sm., which has also been applied for arctic plants, should be used for central Asian plants only, and mention several characters that distinguish the two taxa. CoL16 and ITIS, however, do not accept the name *R. arcticus* but advocate the name *R. pedatifidus* var. *affinis* (R. Br.) L. D. Benson to replace *R. affinis.* Their reasons for treating the arctic and northern plants as *R. pedatifidus* var. *affinis* are, however, not apparent, and we therefore accept the name *Ranunculus arcticus* for Greenlandic plants.

### Ranunculus sulphureus Sol.

We maintain the name *Ranunculus sulphureus* that was used by Böcher et al. (1978), following FCAA, CoL16 and ITIS. In PAF the species is treated as *Ranunculus sulphureus* var. *sulphureus* Sol. It is noted that “In addition to the mainly arctic var. *sulphureus* there is a non-arctic var. *intercedens* Hultén in Kamtchatka and the Aleutian Islands.”

### Saxifraga cespitosa L.

The name *Saxifraga caespitosa* used by Böcher (1978) is an orthographic variant (misspelling) for *Saxifraga cespitosa* (ITIS).

### Saxifraga platysepala (Trautv.) Tolm

We maintain the name *Saxifraga platysepala* as used by Böcher (1978), following PAF, FCAA and CoL16. ITIS, in contrast, accepts the name *Saxifraga flagellaris* ssp. *platysepala* (Trautv.) A.E. Porsild for the taxon. This name was used by Porsild (1964) and Porsild and Cody (1980), but has apparently not been widely adopted. We therefore accept the name used in most databases.

### Silene involucrata (Cham. & Schltdl.) Bocquet, Silene sorensenis (B. Boivin) Bocquet and Silene uralensis ssp. arctica (Th. Fr.) Bocquet

We change the names of the three species included in the genus *Melandrium* by Böcher et al. (1978), following PAF, FCAA, CoL16 and ITIS. Elven and Petrovsky (in PAF) argue that “Molecular-cladistic analyses of the genera of subfam. *Silenoideae* – summarized by Oxelman et al. (2001) – have confirmed some long-time suspicions. *Lychnis* and *Silene* as traditionally circumscribed are polyphyletic or paraphyletic. There is one major monophyletic *Silene* branch that includes most parts of traditional *Silene* and also the previous segregates of, e.g., *Melandrium*, *Gastrolychnis*, and the *Lychnis sibirica* group.”

The name for *Melandrium affine* in *Silene* is *S. involucrata* as applied by Bocquet (1967). The reason for this is according to Elven, Murray, and Petrovsky (in PAF) that “*Agrostemma involucratum* (Cham. & Schltdl.) G. Don 1831 predates the other early combinations with *"furcata"* 1840, *"affinis"* 1842, *"angustiflora"* 1845, and *"vahlii"* 1845.”

The name for *Melandrium triflorum* in *Silene* is according to Elven, Murray, and Petrovsky (in PAF) *S. sorensenis* as applied by Bocquet (1967) because the older epithet *"triflora"* is inapplicable within Silene (homonymy).”

The name for *Melandrium apetalum* ssp. *arcticum* in *Silene* is according to PAF and FCAA *Silene uralensis* ssp. *arctica.* CoL16 and ITIS treat *Silene uralensis* ssp. *arctica* as a synonym for *Silene* *uralensis* ssp. *uralensis* (Rupr.) Bocquet. Elven, Murray, and Petrovsky (in PAF) disagree with Bocquet's view that “subsp. *arctica* is a local Svalbard plant, whereas all the other arctic plants belong to subsp. *uralensis*.” They have compared Svalbard plants with plants from arctic Russia, Greenland, arctic Canada and Alaska, and in the field in several regions. They “can find no differences among these high-arctic circumpolar plants and consider them one uniform taxon. The only epithet relevant for this taxon is *"arctica"*.” The basionym for *Silene uralensis* is *Gasterclychnis uralensis* Rupr. 1845.

### Taraxacum arcticum (Trautv.) Dahlst. aggregate

We maintain the name *Taraxacum arcticum* used by Böcher et al. (1978), and use it as an aggregate, following PAF and FCAA. According to ITIS *Taraxacum* *arcticum* (Trautv.) Dahlst. is a synonym for *Taraxacum hyparcticum* Dahlst but we have however not been able to find the argumentation behind this replacement.

### Vaccinium uliginosum L.

We maintain the name *Vaccinium uliginosum* used by Böcher et al. (1978), following PAF, CoL16 and ITIS. Alsos and Elven (PAF) note that “there is a correlated variation in the very widely distributed *Vaccinium uliginosum*, in morphology, ploidy levels, and distribution patterns. This variation merits some taxonomic recognition.” According to Böcher (1978) most Greenlandic plants belong to subspecies “*microphyllum”* and only some plants might belong to a southern subspecies “*uliginosum*”.

**Table S2.** Number of plots and plot groups were each species occurred.

| **Species** | **No of plot groups** | | **No of plots** | |  | | **Species** | **No of plot groups** | | | **No of plots** | |
| --- | --- | --- | --- | --- | --- | --- | --- | --- | --- | --- | --- | --- |
| *Alopecurus borealis* | 16 | 63 | |  | | *Juncus triglumis* | | | 9 | 31 | |  |
| *Arctagrostis latifolia* | 17 | 61 | |  | | *Kobresia myosuroides* | | | 43 | 170 | |  |
| *Arenaria pseudofrigida* | 18 | 65 | |  | | *Kobresia simpliciuscula* | | | 7 | 23 | |  |
| *Armeria scabra* | 3 | 10 | |  | | *Luzula confusa* | | | 23 | 88 | |  |
| *Arnica angustifolia* | 9 | 23 | |  | | *Luzula nivalis* | | | 13 | 40 | |  |
| *Bistorta vivipara* | 44 | 223 | |  | | *Melandrium* sp. | | | 20 | 40 | |  |
| *Calamagrostis purpurascens* | 4 | 10 | |  | | *Micranthes nivalis* | | | 16 | 35 | |  |
| *Campanula uniflora* | 14 | 49 | |  | | *Minuartia biflora* | | | 5 | 10 | |  |
| *Carex bigelowii* | 11 | 32 | |  | | *Oxyria digyna* | | | 10 | 20 | |  |
| *Carex capillaris* | 16 | 54 | |  | | *Papaver radicatum* | | | 30 | 66 | |  |
| *Carex fuliginosa* | 13 | 36 | |  | | *Pedicularis flammea* | | | 9 | 31 | |  |
| *Carex maritima* | 8 | 12 | |  | | *Pedicularis hirsuta* | | | 15 | 48 | |  |
| *Carex nardina* | 24 | 72 | |  | | *Physaria arctica* | | | 4 | 13 | |  |
| *Carex rupestris* | 28 | 109 | |  | | *Poa arctica* | | | 21 | 57 | |  |
| *Carex supina* ssp. *spaniocarpa* | 4 | 11 | |  | | *Poa glauca* | | | 35 | 155 | |  |
| *Cassiope tetragona* | 13 | 32 | |  | | *Polemonium boreale* | | | 8 | 12 | |  |
| *Cerastium arcticum* | 42 | 160 | |  | | *Potentilla arenosa* | | | 13 | 31 | |  |
| *Chamerion latifolium* | 6 | 19 | |  | | *Potentilla hyparctica* | | | 14 | 51 | |  |
| *Draba arctica* | 11 | 20 | |  | | *Potentilla nivea* | | | 20 | 46 | |  |
| *Draba fladnizensis* | 4 | 12 | |  | | *Potentilla rubricaulis* | | | 12 | 32 | |  |
| *Draba glabella* | 8 | 22 | |  | | *Rhododendron lapponicum* | | | 7 | 15 | |  |
| *Draba subcapitata* | 10 | 27 | |  | | *Rumex acetosella* | | | 10 | 33 | |  |
| *Dryas* sp. | 45 | 228 | |  | | *Sabulina rubella* | | | 23 | 51 | |  |
| *Equisetum arvense* | 15 | 45 | |  | | *Sabulina stricta* | | | 7 | 13 | |  |
| *Equisetum variegatum* | 16 | 31 | |  | | *Salix arctica* | | | 47 | 259 | |  |
| *Eriophorum triste* | 12 | 33 | |  | | *Saxifraga cernua* | | | 39 | 125 | |  |
| *Euphrasia frigida* | 5 | 14 | |  | | *Saxifraga cespitosa* | | | 9 | 16 | |  |
| *Festuca brachyphylla* | 14 | 34 | |  | | *Saxifraga hirculus* | | | 4 | 10 | |  |
| *Festuca hyperborea* | 30 | 93 | |  | | *Saxifraga oppositifolia* | | | 31 | 120 | |  |
| *Festuca rubra* | 10 | 22 | |  | | *Silene acaulis* | | | 38 | 163 | |  |
| *Festuca vivipara* | 10 | 21 | |  | | *Silene involucrata* | | | 7 | 20 | |  |
| *Hierochloë alpina* | 9 | 35 | |  | | *Stellaria longipes* | | | 31 | 112 | |  |
| *Juncus biglumis* | 14 | 37 | |  | | *Trisetum spicatum* | | | 21 | 47 | |  |
| *Juncus castaneus* | 5 | 17 | |  | | *Vaccinium uliginosum* | | | 15 | 43 | |  |

## Supporting References

Al-Shehbaz, I.A. & O’Kane, S. L., J. (2002) Lesquerella is united with Physaria (Brassicaceae). *Novon*, **12**, 319–329.

Allred, K.W. & Barkworth, M.E. (2007) Anthoxanthum L. *Flora of North America north of Mexico. 24. Magnoliophyta: Commelinidae (in part): Poaceae, part 1* (ed Flora of North America Editorial Committee), pp. 758–764.

Böcher, T.W., Fredskild, B., Holmen, K. & Jakobsen, K. (1978) *Grønlands Flora*, 3rd editio. P. Haase & Søns Forlag, Copenhagen.

Brooks, R.E. & Clemants, S.E. (2000) Juncus Linnaeus. *Flora of North America north of Mexico. 22. Magnoliophyta: Alismatidae, Arecidae, Commelinidae (in part), and Zingiberidae* (ed Flora of North America Editorial Committee), pp. 211–255.

Brouillet, L. & Elvander, P.E. (2009) *Micranthes* Haworth. *Flora of North America north of Mexico. 8. Magnoliophyta: Paeoniaceae to Ericaceae* (ed Flora of North America Editorial Committee), pp. 49–70.

Brouillet, L. & Gornall, R. (2007) New combinations in *Micranthes* (a segregate of *Saxifraga*, Saxifragaceae) in North America. *J. Bot. Res. Inst. Texas*, **1**, 1019–1022.

Brysting, A.K., Aiken, S.G., Lefkovitch, L.P. & Boles, R.L. (2003) *Dupontia* (Poaceae) in North America. *Canadian Journal of Botany*, **81**, 769–779.

Brysting, A.K., Fay, M.F., Leitch, I.J. & Aiken, S.G. (2004) One or more species in the arctic grass genus *Dupontia*? – a contribution to the Panarctic Flora project. *Taxon*, **53**, 365–382.

Cody, W.J. (1994) Nomenclatural changes and new taxa for the Yukon flora. *Canadian Field-Naturalist*, **108**, 93–95.

Darbyshire, S.J. & Pavlick, L.E. (2007) *Festuca* L. *Flora of North America north of Mexico. 24. Magnoliophyta: Commelinidae (in part): Poaceae, part 1* (ed Flora of North America Editorial Committee), pp. 389–443.

Dillenberger, M.S. & Kadereit, J.W. (2014) Maximum polyphyly: Multiple origins and delimitation with plesiomorphic characters require a new circumscription of Minuartia (Caryophyllaceae). *Taxon*, **63**, 64–88.

Eidesen, P.B. (2007) The unfaithful birches – what is a species in phylogeography? *Paper in: Eidesen, P. B., Arctic–alpine plants on the move – Individual and comparative phylogeographies reveal responses to climate change. – Dr. Sci. thesis, Univ. Oslo, Oslo.*

Ekman, E. (1933) Contribution to the *Draba* flora of Greenland. V. *Svensk Botanisk Tidsskrift*, **27**, 97–103.

Elven, R. & Elvebakk, A. (1996) Vascular plants. *A Catalogue of Svalbard Plants, Fungi, Algae, and Cyanobacteria. Part 1.* (eds A. Elvebakk), & P. Prestrud), p. 198: 9-55. Norsk Polarinst. Skr.

Harris, J.G. (1985) *A Revision of the Genus* Braya *(Cruciferae) in North America*. University of Alberta, Edmonton.

Harris, J.G. (2010) *Braya* Sternberg & Hoppe. *Flora of North America north of Mexico. 7. Magnoliophyta: Salicaceae to Brassicaceae* (ed Flora of North America Editorial Committee), pp. 546–552.

Hijmans, R.J., Cameron, S.E., Parra, J.L., Jones, P.G. & Jarvis, A. (2005) Very high resolution interpolated climate surfaces for global land areas. *International Journal of Climatology*, **25**, 1965–1978.

Holub, J. (1972) Folia Geobotanica & Phytotaxonomica. *Folia Geobotanica Phytotax.*, **7**, 81–90.

Hultén, E. (1943) Flora of Alaska and Yukon. III. Monocotyledoneae (Liliiflorae, Microspermae) Dicotyledoneae (Salicales). *Acta Univ. Lund., n. s., sect. 2, 39, 1* pp. 413–567.

Hultén, E. (1956) The *Cerastium alpinum* complex. A case of world-wide introgressive hybridization. *Svensk Botanisk Tidskrift*, **50**, 411–495.

Hultén, E. (1968) *Comments on the Flora of Alaska and Yukon*.

Hylander, N. (1945) Nomenklatorische und systematische Studien über nordische Gefässpflanzen. *Uppsala Universitet Årsskrift*, **7**, 337.

Hylander, N. (1953) *Nordisk Kärlväxtflora, I*. lmqvist & Wiksell, Stockholm.

Kirschner, J. (2001) Proposals to reject the names *Juncus cymosus*, *J. radicans*, *Luzula capillaris*, *L. hyperborea, L. interrupta*, and *Rostkovia brevifolia* (Juncaceae). *Taxon*, **50**, 1193–1197.

Lammers, T.G. (2007) *World Checklist and Bibliography of Campanulaceae. IX*. Roylal Botanic Gardens, Kew.

Lefèbvre, C. & Vekemans, X. (1994) Morphological variation, taxonomy and evolution of *Armeria maritima* (Plumbaginaceae) from North America. *American Journal of Botany*, **81 (suppl.**, 167.

Müntzing, A. (1932) Apomictic and sexual seed formation in *Poa*. *Hereditas (Lund)*, **17**, 131–154.

Müntzing, A. (1940) Further studies on apomixis and sexuality in *Poa*. *Hereditas (Lund)*, **26**, 115–190.

O’Kane Jr., S.L. (2010) *Physaria* (Nuttall ex Torr. & A. Gray) A. Gray. *Flora of North America north of Mexico. 7. Magnoliophyta: Salicaceae to Brassicaceae* (ed Flora of North America Editorial Committee), pp. 616–665.

Oxelman, B., Lidén, M., Rabeler, R.K. & Popp, M. (2001) A revised classification of the tribe Sileneae (Caryophyllaceae). *Nordic Journal of Botany*, **20**, 513–518.

Polunin, N. (1940) Botany of the Canadian Eastern Arctic. Part I. Pteridophyta and Spermatophyta. *Bull. Natl. Mus. Canada*, **92**, 408.

Porsild, A.E. (1964) Illustrated flora of the Canadian Arctic Archipelago. *Natl. Mus. Can. Bull.*, **146**, 218.

Porsild, A.E. & Cody, W.J. (1980) Vascular plants of the continental Northwest Territories, Canada. *National Museum of Natural Sciences, Canada*, 667.

Rollins, R.C. (1993) *The Cruciferae of Continental North America*. Stanford University Press, Stanford.

Schouten, Y. & Veldkamp, J.F. (1985) A revision of Anthoxanthum including Hierochloë (Gramineae) in Malesia and Thailand. *Blumea*, **30**, 319–351.

Shetler, S.G. (1982) Variation and evolution of nearctic harebells (Campanula subsect. Heterophylla). *Phanerogamarum monographiae 11* p. 516. Cramer, Vaduz.

Soltis, D.E., Kuzoff, R.K., Conti, E., Gornall, R. & Ferguson, K. (1996) *mat*K and *rbc*L gene sequence data indicate that Saxifraga (Saxifragaceae) is polyphyletic. *American Journal of Botany*, **83**, 371–382.

Soltis, D.E., Kuzoff, R.K., Mort, M.E., Zanis, M., Fishbein, M., Hufford, L., Koontz, J. & Arroyo, M.K. (2001) Elucidating deep-level phylogenetic relationships in Saxifragaceae using sequences for six chloroplastic and nuclear DNA regions. *Annals of the Missouri Botanical Garden*, **88**, 669–693.

Sommerfelt, S.C. (1833) Bidrag til Spitsbergens og Beeren-Eilands Flora, efter Herbarier medbragte af M. Keilhau. *Magazin for Naturvidenskaberne*, **11**, 232–252.

Soreng, R.J. (2003) *Alopecurus*. *Catalogue of New World Grasses (Poaceae): IV. Subfamily Pooideae, Contr. U.S. Natl. Herb. Smithsonian Institution, Washington, D.C.* (eds R.J. Soreng),, P.M. Peterson),, G. Davidse),, E.J. Judziewicz),, F.O. Zuloaga),, T.S. Filgueiras), & O. Morrone), p. 48: 97-106.

Soreng, R.J., Peterson, P.M., Davidse, G., Judziewicz, E.J., Zuloaga, F.O., Filgueiras, T.S. & Morrone, O. (2003) Catalogue of New World grasses (Poaceae): IV. Subfamily Pooideae. *Contributions from the United States National Herbarium*, **48**, 730.

Tzvelev, N.N. (1976) *Zlaki SSSR*. Nauka, Leningrad.

Warwick, S.I., Al-Shehbaz, I.A., Sauder, C.A., Harris, J.G. & Koch, M. (2003) Phylogeny of *Braya* and *Neotorularia* (Brassicaceae) based on nuclear ribosomal internal transcribed spacer and chloroplast *trn*L intron sequences. *Canadian Journal of Botany*, **82**, 376–392.
